# Advances on the Visualization of the Internal Structures of the European Mistletoe: 3D Reconstruction Using Microtomography

**DOI:** 10.3389/fpls.2021.715711

**Published:** 2021-09-20

**Authors:** Max D. Mylo, Mara Hofmann, Alexander Delp, Ronja Scholz, Frank Walther, Thomas Speck, Olga Speck

**Affiliations:** ^1^Plant Biomechanics Group, Botanic Garden Freiburg, University of Freiburg, Freiburg, Germany; ^2^Cluster of Excellence livMatS @ FIT – Freiburg Center for Interactive Materials and Bioinspired Technologies, Freiburg, Germany; ^3^Department of Materials Test Engineering (WPT), TU Dortmund University, Dortmund, Germany

**Keywords:** *Viscum album*, haustorial system, sinkers, hemiparasite, stem parasite, X-ray microtomography (microCT), biased sex ratio

## Abstract

The European mistletoe (*Viscum album*) is a dioecious epiphytic evergreen hemiparasite that develops an extensive endophyte enabling the absorption of water and mineral salts from the host tree, whereas the exophytic leaves are photosynthetically active. The attachment mode and host penetration are well studied, but little information is available about the effects of mistletoe age and sex on haustorium-host interactions. We harvested 130 plants of *Viscum album* ssp. *album* growing on host branches of *Aesculus flava* for morphological and anatomical investigations. Morphometric analyses of the mistletoe and the (hypertrophied) host interaction site were correlated with mistletoe age and sex. We recorded the morphology of the endophytic systems of various ages by using X-ray microtomography scans and corresponding stereomicroscopic images. For detailed anatomical studies, we examined thin stained sections of the mistletoe-host interface by light microscopy. The diameter and length of the branch hypertrophy showed a positive linear correlation with the age of the mistletoe. Correlations with their sex were only found for ratios between host branch and hypertrophy size. A female bias of about 76% was found. In a 4-year-old mistletoe, several small, almost equally sized sinkers and the connected cortical strands extend over more than 5 cm within the host branch. In older mistletoes, one main sinker was predominant and occupied an increasingly large proportion of the stem cross-section. Bands of vessels ran along the axis of the wedge-shaped haustoria and sinkers and bent sideways toward the mistletoe-host interface. At the interface, the vascular elements of the host wood changed their direction and formed vortices near the haustorium.

## Introduction

The genus *Viscum* is now assigned to the family Santalaceae, which belongs to the order Santalales (Stevens, 2001 onward; Su et al., 2015; Nickrent, 2020), but in other family delimitations it was placed within the Viscaceae (Nickrent et al., 2010 and several others before). One of its nearly 100 species is the European mistletoe, *Viscum album* L., which is native to Europe and western and southern Asia. But it is also locally distributed in the United States as a neophyte. From the region around Sebastopol in California, where it was introduced around 1,900 by horticulturist Luther Burbank (Scharpf and Hawksworth, 1976; Hawksworth and Scharpf, 1986), it is spreading steadily and is already infesting at least 25 host species causing problems for orchards and urban hardwoods (Shaw and Lee, 2020). As an epiphytic hemiparasite, it develops an endophytic haustorial system of sinkers that enable the absorption of water and mineral salts from the stems of the host tree, whereas its exophyte is still capable of photosynthesis (Zuber, 2004). These perennial and evergreen plants are dioecious, with male plants bearing staminate flowers and female plants bearing carpellate flowers and white or yellow berries (Kuijt, 1969). Four subspecies are known in Europe and differ from each other by host specificity and minor morphological characteristics such as leaf shape and size, the color of their berries, and the viscosity of their berry mucus (Grundmann et al., 2014). *Viscum album* ssp. *album* has white berries and grows exclusively on angiosperms (e.g., *Aesculus*, *Malus, Populus*, and *Tilia*). *Viscum album* ssp. *abietis* also has white berries but grows exclusively on *Abies* species. *Viscum album* ssp. *austriacum* bears yellow berries and parasitizes mainly *Pinus* species but sometimes also colonizes *Larix* and *Picea* species. *Viscum album* ssp. *creticum* develops white fruits and grows exclusively on *Pinus halepensis* ssp. *brutia* on Crete (Grundmann et al., 2014).

**FIGURE 1:**
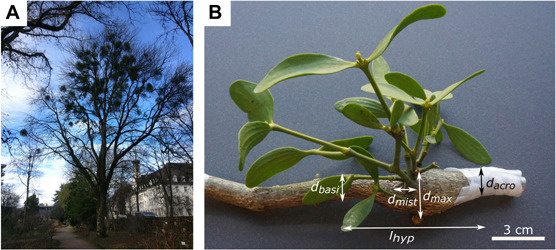
The mistletoe *Viscum album* ssp. *album* parasitizing its host tree *Aesculus flava*. **(A)** Host tree in the Botanic Garden Freiburg, Germany, carrying mistletoe plants of various ages. **(B)** Hypertrophic host branch (brown) and a 5-year-old mistletoe (green). The maximum diameter of host hypertrophy (*d*_*max*_), branch diameter acropetally to hypertrophy (*d*_*acro*_), branch diameter basipetally to hypertrophy (*d*_*basi*_), diameter of mistletoe attachment (*d*_*mist*_), and length of hypertrophy (*l*_*hyp*_) are marked.

*Viscum album* forms large plants that, over 20 years, can reach diameters of about 2.5 m (Heide-Jørgensen, 2008; Figure 1A). The age of a mistletoe plant can be easily determined because of its regular growth (Nierhaus-Wunderwald and Lawrenz, 1997; Figure 1B). In its first year, the mistletoe grows solely inside the host. The shoot develops in the second year and forms a node every year thereafter. In its fourth year, the shoot branches for the first time and, from then on, repeatedly forms a new branching point every year. The plant starts flowering at about 5 years and is mainly pollinated by insects. Wind pollination plays a minor role. Its single-seeded berries are a food source for birds in winter (Ramm et al., 2000). Mistle thrushes (*Turdus viscivorus*) and blackcaps (*Sylvia atricapilla*) are especially important for the European mistletoe, as they are the main animal dispersers of its seeds. The birds eat and excrete the seeds or destroy the berry exocarp with their beak thereby exposing the seed and allowing its growth. The mesocarp, which is sticky because of the viscin that it contains, attaches the seed to the host branch (Horbelt et al., 2019). In spring, the germination process is induced by the rising temperatures and light intensity, and the parasite starts to infest its host and form its haustorium. Chemical substances from the host play no role as triggers of germination (Nierhaus-Wunderwald and Lawrenz, 1997).

Three main stages can be distinguished in haustorium development: (i) the formation of the holdfast, (ii) the development of the intrusive organ, and (iii) the connection of the haustorium to the host (Heide-Jørgensen, 2008; Teixeira-Costa, 2021). After excretion by the bird and attachment by viscin to the host, the embryo forms a hypocotyl that, driven by negative phototropism and geotropism, bends toward the host branch (Thoday, 1951). When the tip of the hypocotyl touches the host, the physical contact stimulus induces its flattening. The epithelium produces lipidic glue, which adheres to the host surface, and forms a holdfast. The host periderm is degraded (probably by means of enzymes), and the parasite penetrates the host cortex. Subcortical cells of the holdfast collapse, and the epithelial cells form papillae that grow into the host and remove layers of cortical tissue. When the intrusive organ reaches the host cambium, the formation of the haustorial meristem is induced, and active penetration stops. Instead, the haustorial meristem produces tissue simultaneously with the host cambium (Thoday, 1951). With each new annual ring of the branch, the sinker also grows and thus forms its typical wedge shape. Bands of vessel elements run from the meristem through the sinker parenchyma toward the tip of the sinker and bend sideward connecting with host xylem. Haustoria do not penetrate the host xylem; they are simply embedded in host xylem tissue (Zuber, 2004). At the interface of the host wood and mistletoe haustorium, vessels of the mistletoe form an open connection to vessel elements of the host (Smith and Gledhill, 1983). The identity of the haustorium organ has anatomical similarities to both roots and shoots but also exhibits decisive differences from each; for this reason, the haustorium is now referred to as a root-shoot mosaic (Teixeira-Costa, 2021).

In addition to the sinkers, the mistletoe forms cortical strands that run along the host branch (Heide-Jørgensen, 2008). Occasionally, these strands induce new shoots, but mostly, they produce secondary sinkers. The latter are probably induced by the exogenous impact of the host, because they form when cortical strands are located close to the host cambium (Sallé, 1978). Infections by the parasite can lead to host wood hypertrophy that occurs, in particular, basipetally from the infection site (Smith and Gledhill, 1983). Other reactions of the host to the attack of the mistletoe are the accumulation of polyphenols in the phellem tissue, phellem lignification, and an increase in fiber cells (Grundmann et al., 2014).

Although much is known about the anchorage and penetration of the host by the European mistletoe, little information is available on the growth and further development of the endophyte as it ages. The aim of this project has therefore been to gain a deeper understanding of the connection between the European mistletoe haustorium and the host branch by (i) determination of the influence of mistletoe age and sex on the morphometry of the connection and (ii) investigation of the morphological (including age dependency) and anatomical characteristics of the interface. Our study focuses on the three-dimensional analysis of the interface between two different structural materials (Wegst et al., 2015) that we have quantified morphometrically and have visualized by various imaging techniques. We have performed morphometric studies to investigate possible age or sex dependencies of branch hypertrophy and of the host-parasite interface. The morphology of the haustorial system has been visualized by X-ray microtomography (microCT) scans of three specimens of different ages and complemented by stereomicroscopic images and tissue segmentation. For anatomical visualization, we have observed stained sections of the mistletoe-host interface and studied them at the cellular level via light microscopy.

## Materials and Methods

### Plant Material

Host branches carrying plants of the European mistletoe (*Viscum album* ssp. *album* L.; hereafter *V. album*) were cut from one tree of the species *Aesculus flava* Sol. (hereafter *A. flava*) located at the Botanic Garden of the University of Freiburg (Germany) on May 18, June 22 and August 5, 2020 (Figure 1A).

### Determination of Sex, Age, and Morphometry

Since younger plants lack flowers and berries, sex could only be determined for mistletoes of 6 years and older. If this was not possible, the plants were classified as juvenile. Age was determined by counting nodes starting with 2 years at the first node, or (if not visible) with 4 years at the first branching. Branch diameters of the host tree were measured at the widest point of the hypertrophy (*d*_*max*_), acropetally from the hypertrophy (*d*_*acro*_), and basipetally from the hypertrophy (*d*_*basi*_), and of the mistletoe branch directly at the attachment to its host (*d*_*mist*_) by using a digital caliper (Mitutoyo Absolute Digimatic, measuring accuracy: ±0.03 mm, Kawasaki, Japan). Since the branches were not expected to have a perfectly circular geometry, diameters were measured twice and, if possible, orthogonally offset, and the arithmetic mean was determined. The length of the hypertrophy (l*_*hyp*_*) was measured using a measuring tape (Figure 1B and Supplementary Table 1).

### Morphological Analyses by microCT

Three mistletoe samples representing different age groups were selected for morphological analysis. The ends of cut sections were sealed with Parafilm (Bemis Company, Neenah, WI, United States) to prevent quick dehydration. A 4-year-old sample was scanned with a resolution of 10 μm, by using a SKYSCAN 1272 microCT in combination with SKYSCAN software (version 1.1.10, both Bruker Corporation, Billerica, MA, United States). The batch scan technique, in which three individual scans along the branch axis are merged into one overview scan, was performed. For further processing of the scans, the data were reconstructed using NRecon software (version 1.6.10.1, Micro Photonics Inc., Allentown, PA, United States). Data were visualized using Avizo software (version 2020.2, Thermo Fisher Scientific, Waltham, MA, United States).

The 8- and 17-year-old samples were scanned at a resolution of 86 μm using a scanner with a larger test chamber volume (Nikon XT H 160) and corresponding software (Inspect X, XT 4.4.2). The data reconstruction was performed using CT-Pro 3D software (version XT 4.4.2, all Nikon, Chiyoda, Japan), and data visualization and video creation were performed with VGStudio MAX software (version 2.2.4, Volume Graphics GmbH, Heidelberg, Germany). Further details of the samples and the related scanning and reconstruction settings can be found in Supplementary Table 2.

After being scanned, the 4-year-old sample was cut by hand into transverse sections of one millimeter with a razor blade, and pictures of the sections were taken via a stereomicroscope (SZX9, Olympus K. K., Shinjuku, Tokyo, Japan) in combination with a Color View II camera (Soft Imaging System GmbH, Münster, Germany) and the corresponding software (Cell^D version 2.6, Olympus K. K., Shinjuku, Tokyo, Japan). These recordings were used manually to segment the mistletoe and host tissue based on the gray scale data of all 5,402 obtained slices and, especially, to distinguish host cortex from mistletoe sinkers and cortical strands. The tissue segmentation and the presentation of the segmented data were carried out using Avizo software. To keep the data size manageable, the mistletoe scan was split into 16 individual datasets that were reduced to 500,000 nodes each and later reassembled.

### Anatomical Analyses by Light Microscopy

**FIGURE 2:**
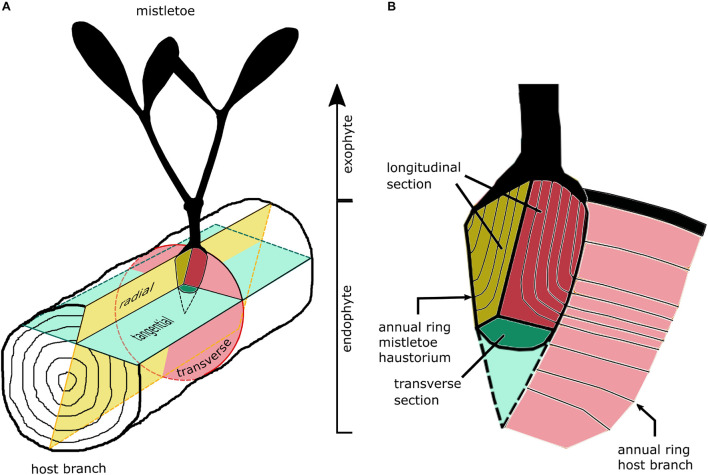
Sketches of the section planes of **(A)** host branch and **(B)** the primary sinker of the mistletoe haustorium in the host wood.

Histological sections were prepared from host tree blocks that contained the mistletoe sinkers. The blocks were sawn by hand to a size suitable for light microscopic analysis. The mistletoe plants used for the anatomical studies were between 7 and 19 years old. Figure 2 shows the section planes of the host branch and the primary sinker of the mistletoe. In the case of the mistletoe sinker, we summarized the tangential and radial section planes as the longitudinal plane leading to following combinations: (i) transverse host plane and longitudinal haustorium plane, (ii) radial host plane and longitudinal haustorium plane, and (iii) tangential host plane and transverse haustorium plane.

Samples for microtome sectioning were softened for 4 days at 60 °C in 10% ethylene diamine and subsequently embedded in Polyethylenglycol 2000, a method slightly modified according to Barbosa et al. (2010) (instead of a dehydration series, the water of a 25% PEG solution was allowed to evaporate slowly). Thin sections with a thickness of 10 μm were cut using a rotary microtome (HistoCore BIOCUT, Leica Biosystems Nussloch GmbH, Nussloch, Germany). 0.05% [w/v] toluidine blue O (TBO) was used for overview staining, highlighting lignified tissue in blue-green and non-lignified tissue in red-purple. TBO images were captured under bright-field conditions by a Zeiss Axio Observer Inverted microscope equipped with a microscope camera (Axiocam 506 color) and Zen 2.6 pro software (version 6.1.7601) (all Carl Zeiss AG, Oberkochen, Germany). 1% [w/v] acridine orange staining (ACO) revealed lignified tissue in bright yellow/orange, whereas non-lignified tissue was stained dark brown/red. ACO images were captured with a light microscope (BX61) equipped with an FITC filter and a DP71 camera and Cell^P software (2.6, all Olympus Soft Imaging Solutions GmbH, Shinjuku, Tokyo, Japan).

Sections were also cut by hand from fresh material by using a razor blade. Phloroglucinol (5% [w/v] EtOH + HCl, PHO) was used to stain lignified tissues in red, whereas non-lignified tissues remain unstained. We examined the sections with a Primo Star microscope equipped with an Axiocam ERc 5s camera (both Carl Zeiss AG, Oberkochen, Germany).

### Statistics

Morphometric raw data and descriptive statistics were recorded and analyzed with Excel (version 2016, Microsoft Corporation, Redmond, WA, United States). Because of the non-normal distribution of the groups, all data are described using the median with the respective interquartile ranges (IQR). Further statistical analyses were performed using the statistical software GNU R v.4.0.4 (R Core Team, 2019), including the packages “car” (Fox and Weisberg, 2019), “ggpubr,” and “stats.” After checks for normally distributed data (Shapiro–Wilk test) and homoscedasticity (Levene test), paired datasets of the branch diameters from the various samples were tested for significant differences by using the non-parametric Friedman test with a subsequent Holm correction for post-hoc testing. A significance level of α = 0.05 was used for all statistical tests. Statistical details and results are presented in Supplementary Table 3.

We tested the morphometric variables for rank correlation (Spearman’s rank correlation coefficient ρ) and linear correlation (Pearson’s correlation coefficient ρ and coefficient of determination *R*^2^) with age. The sign of the Spearman’s ρ indicates the direction (positive or negative) of the correlation. The absolute value of the Spearman’s ρ classifies the strength of the correlation, with |ρ| < 0.3 indicating no correlation, 0.3 ≤ |ρ| < 0.5 indicating a weak correlation, 0.5 ≤ |ρ| < 0.7 indicating a medium correlation, and |ρ| ≤ 0.7 indicating a strong correlation (adapted from Kampowski et al., 2017).

Multiple linear regressions were performed to test for the influence of sex (juvenile, female, and male) on the variables, in addition to the influence of age. A *p*-value smaller than 0.05 indicates that the sex predictor makes a significant contribution to the linear regression model.

## Results

### Sex, Age, and Morphometry of the Mistletoes

#### Sex and Age of the Mistletoes

**FIGURE 3:**
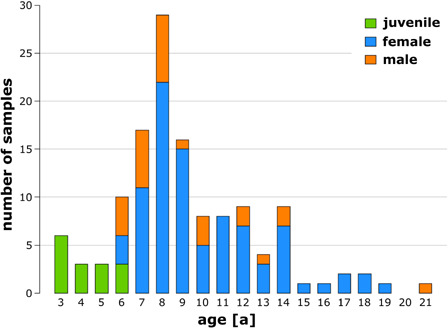
Number of analyzed juvenile (green), female (blue), and male (orange) European mistletoe plants sorted by age.

A total of 130 mistletoe plants, all harvested from one host tree, were examined, of which 15 were juvenile (3–6 years old), 88 were female (6–19 years old), and 27 were male (6–21 years old). Figure 3 shows the number of samples sorted by their age and sex. Detailed raw data can be found in Supplementary Table 1.

#### Size of Host Branch Hypertrophy in Haustorial Region

Size measurements were carried out as shown in Figure 1B. Table 1 gives the absolute values for the diameter of the mistletoe at the attachment, the host branch diameters, the host branch hypertrophy length, and the calculated morphometric ratios for the haustorial region. The values in Table 1 are based on all available data without discrimination by sex or age of the samples. Because of incomplete hypertrophic regions or abnormal branch growth, we were unable to determine all the variables for each sample.

**TABLE 1:** Dimensions of host branch hypertrophy and diameter of mistletoe attachment.

Variables		*N*	Med	IQR	Max	Min	*Correlation with age*		
							Spearman’s ρ	Pearson’s ρ	Pearson *R*^2^
*d*_*max*_	[mm]	129	37.12	20.02	77.62	8.50	0.69	0.70	0.49
*d*_*acro*_	[mm]	128	24.61	16.43	50.49	5.11	0.50	0.47	0.22
*d*_*basi*_	[mm]	126	25.32	17.80	53.97	5.73	0.50	0.48	0.23
*d*_*mist*_	[mm]	104	15.92	16.26	56.85	2.67	0.81	0.78	0.61
*l*_*hyp*_	[mm]	122	11.10	8.85	35.00	1.90	0.66	0.65	0.42
*d*_*max*_⋅*l*_*hyp*_	[mm^2^]	122	397.30	545.53	2608.38	20.40	0.70	0.64	0.40
$\frac{d_{max}}{l_{hyp}}$	[ ]	122	3.23	1.46	8.62	1.56	−0.41	−0.45	0.20
$\frac{d_{max}}{d_{acro}}$	[ ]	128	1.43	0.45	3.56	0.92	0.27	0.24	0.06
$\frac{d_{max}}{d_{basi}}$	[ ]	126	1.40	0.47	3.28	0.93	0.26	0.23	0.05
$\frac{d_{max}}{\left(d_{acro}+d_{basi}\right)/2}$	[ ]	125	1.42	0.38	3.42	0.93	0.29	0.25	0.06

*Median (med), interquartile range (IQR), minimum (min), and maximum (max) values of the maximal (d_max_), acropetal (d_acro_) and basipetal (d_basi_)host branch diameters, length of hypertrophy (l_hyp_), diameter of mistletoe attachment (d_mist_), and various morphometric ratios calculated from these values in the haustorium region are presented. If the diameter was measured in two perpendicular directions, the mean value was used for the calculation. N indicates the sample size. The correlation coefficients of the categories with age were calculated for rank correlations (Spearman’s ρ) and linear correlations (Pearson’s ρ and the coefficient of determination R^2^).*

We found a median increase of the host branch diameter in the area of the haustorial system (*d*_*max*_) compared with the acropetal (*d*_*acro*_) and basipetal (*d*_*basi*_) host branch diameter by factors of about 1.51 and 1.47, respectively. The acropetal diameter was significantly smaller than the basipetal diameter (*p* = 2.7 × 10^–2^), and the maximal diameter was significantly larger than the acropetal diameter (*p* < 1.2 × 10^–21^) and the basipetal diameter (*p* < 2.0 × 10^–21^).

#### Branch Hypertrophy as a Function of Age

Rank and linear correlations of the morphometric dimensions with age were calculated, and the respective ρ values were used to quantify its goodness of fit (Table 1 and Supplementary Table 3). Age dependency was found for several variables. Strong positive rank correlations with age were found for the mistletoe attachment diameter and the product of the maximum host branch diameter and the length of hypertrophy (*d*_*max*_⋅*l*_*hyp*_[mm^2^]), the latter being considered as a representative of the size of the host branch hypertrophy. Medium positive rank correlations were found for maximum, acropetal, and basipetal diameters of the host branch and the length of its hypertrophy. A weak negative rank correlation was found for the quotient of the maximum host branch diameter and the length of hypertrophy $\left(\frac{d_{max}}{l_{hyp}}\left[\right]\right)$, which can be considered as a representative of the compactness of the hypertrophy. All ρ values of the Pearson’s rank test were less than 10% above those of the linear Spearman’s correlation. This indicted that the linear part of the correlation of the data was predominant, and that if a corrleation was present, it could be approximated as being linear.

#### Branch Hypertrophy as a Function of Sex

A significant negative contribution of the predictor sex was found for the female (*p* = 6.3 × 10^–5^) and male (*p* = 1.0 × 10^–4^) mistletoes for $\frac{d_{max}}{l_{hyp}}$, in comparison with the juvenile samples. In addition, the male predictor had a significant positive influence on the variables $\frac{d_{max}}{d_{acro}}$ (*p* = 2.2 × 10^–2^), $\frac{d_{max}}{d_{basi}}$ (*p* = 4.0 × 10^–2^), and $\frac{d_{max}}{\left(d_{acro}+d_{basi}\right)/2}$ (*p* = 2.5 × 10^–2^), whereas the female predictor had no further significant influence. In a comparison between the female and male samples, a significant contribution for the predictor sex was only found for $\frac{d_{max}}{d_{acro}}$ (*p* = 3.1 × 10^–2^).

### Morphology

**FIGURE 4:**
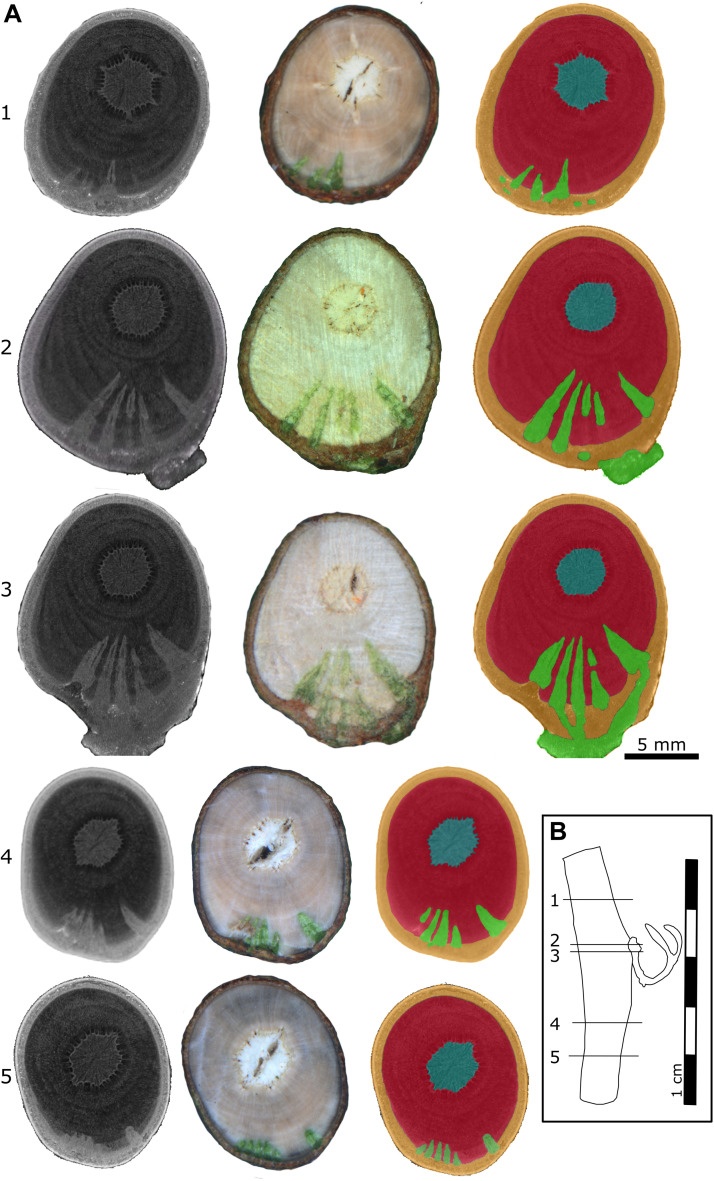
Location of a 4-year-old mistletoe endophyte inside the host branch. **(A)** Transverse sections of the host branch. Images were taken with a microCT (first column) and a stereo microscope (second column). The third column shows the microCT scan recolored by hand according to the stereomicroscopic images. Blue: host pith, red: host wood, orange: host cortex, green: mistletoe. The positions of the individual sections (1–5) are marked in the sample outline in panel **(B)**. **(B)** Outline of the host branch showing the position of the five transverse sections from apical (1) to basal (5). Parts of the exophyte had broken off during cutting, as is visible in its mildly different shape in sections 2 and 3.

X-ray microtomography (microCT) was chosen to visualize the morphology of the mistletoe endophyte at a cellular to tissue level (Landis and Keane, 2010; Teixeira-Costa and Ceccantini, 2016; Hesse et al., 2019). The scans showed high contrast between the host wood, the host pith, and the mistletoe sinkers. However, hardly any difference was found between the host cortex and the mistletoe sinkers and cortical strands, making it difficult to distinguish between these tissues (Figure 4). Nevertheless, in stereomicroscopic images of the transverse sample sections, the mistletoe tissues could be easily identified based on their green color. The scans visualized the extent of the hypertrophy of host wood. In the host branch of a 4-year-old mistletoe, the distance between the host cortex and pith was two to three times larger on the side of the mistletoe shoot than on the opposite side (Figure 4A3).

**FIGURE 5:**
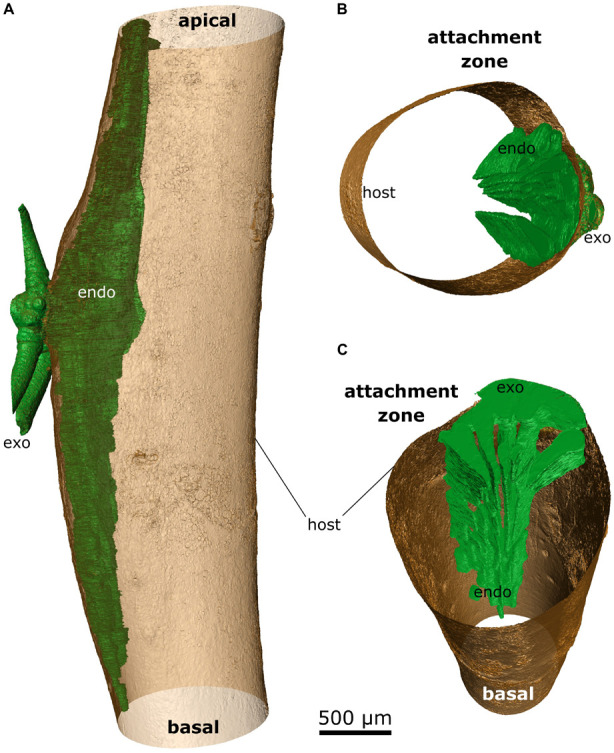
False-color representation of segmented microCT data of the 4-year-old mistletoe. The mistletoe, consisting of endophyte (endo) and exophyte (exo, trimmed to fit the size of the scan volume), is colored green; the outer hull of the host branch is colored brown. **(A)** Overview of the mistletoe-host connection with the sinkers running basally and acropetally through the host branch. Detailed views of **(B)** the attachment site and **(C)** the sinkers running basipetally from the attachment site (the host branch is depicted as being hollow).

Up to five wedge-shaped mistletoe sinkers grew from the infestation site, with their tips pointing toward the center of the host branch and hardly differing in size (Figures 4A, 5B,C). Near the attachment zone, the sinkers connect with the cortical strands, which run basipetally and acropetally trough the host bark. The size of the sinkers decreased the further basally or apically from the attachment zone they were located (Figures 5A,C). In the 4-year-old mistletoe, the sinkers and cortical strands were distributed over a distance of more than 5 cm along the host branch (Figures 4, 5A). Some sinkers were directly aligned with the mistletoe shoot, whereas many others were located around the host branch and connected by cortical strands (Figures 4A, 5).

**FIGURE 6:**
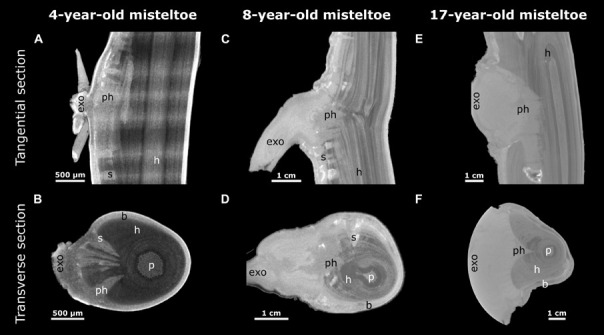
Reconstructed X-ray microtomographic images of mistletoe-host interactions at various mistletoe ages. Tangential **(A,C,E)** and transverse sections (**B**,**D**,**F**; with regard to the host branch) through the attachment site are presented. The mistletoe, divided into exophyte (exo), primary haustorium (ph), sinker (s), and the host branch (h) with its pith (p) and bark (b) can be differentiated. A 4-year-old (**A**,**B**, young), an 8-year-old (**C**,**D**, middle age), and a 17-year-old (**E**,**F**, old) mistletoe are presented as representatives of their age group. The semicircular geometry of the exophyte in panel **(F)** results from the circular restriction of the scan volume.

With increasing age of the mistletoe, the initially rather thin, wedge-shaped sinkers (Figures 6A,B) at the attachment site increasingly thickened and made up a larger part of the entire cross-section. Additional sinkers were visible at this intermediate age, although these were now clearly distinguishable from the main sinker because of their smaller size (Figures 6C,D). In the old sample, only the one main sinker was visible at the level of the attachment. The haustorium formed into a wider wedge (in the cross-section of the host) around which the host wood grew (Figures 6E,F). Such a coalescence of the individual sinker structures also became apparent in the longitudinal view of the host branch (Figures 6A,C,E). Smaller sinkers were visible only basal to the attachment zone and on the opposite side of the host branch, although whether these were structures connected by cortical strands or a new site of infection was unclear (Supplementary Video 3). In addition, the transverse geometry of the infested host branch noticeably changed from approximately oval (young mistletoe) to nearly triangular with a tip at the non-infested side (older mistletoe) (Figures 6B,D,F). Please note that the semicircular geometry of the exophyte of the old mistletoe (F) does not represent its growth habit but results from the size limitations of the scanning. Videos of the reconstructed microCT recordings of all three samples and of the segmented young mistletoe with animations through the host branch axis can be found in the Supplementary Videos 1–4.

### Anatomy

**FIGURE 7:**
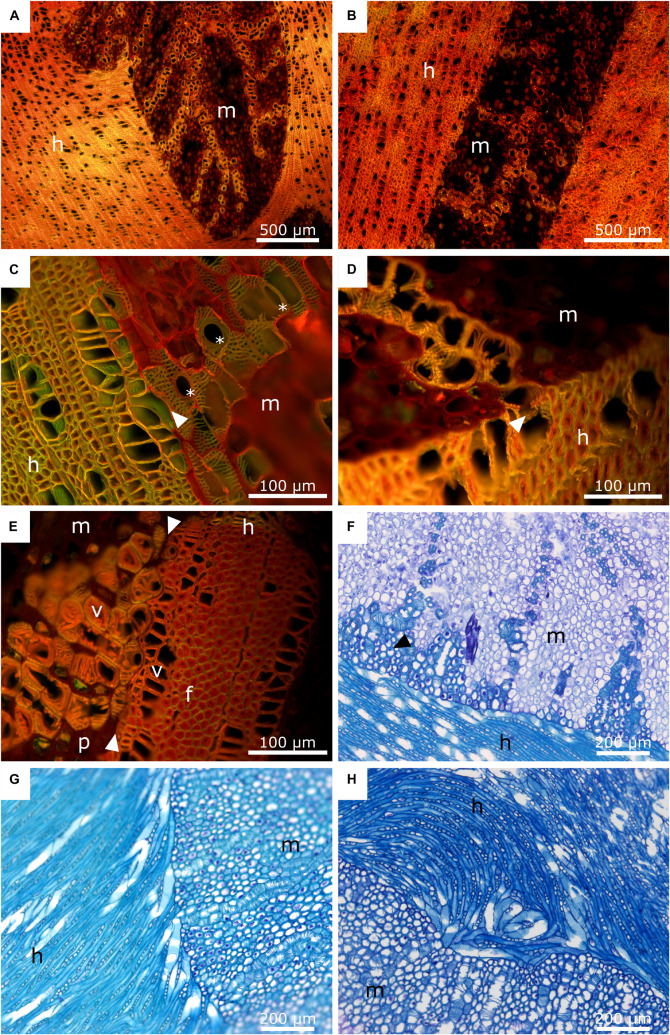
Anatomy of the mistletoe-host interface. The mistletoe haustorium/sinkers and the host wood are marked with “m” and “h”, respectively. **(A,B)** Acridine orange staining of mistletoe sinkers and their host. The sinkers are sectioned longitudinally. The transverse plane of the host wood is shown. **(C,D)** Acridine orange staining of the interface of a mistletoe haustorium (longitudinal) and the host wood (transverse). The black arrows mark the contact area between the vessel elements of the host and the mistletoe tracheids. The asterisks mark perforation plates of the mistletoe vessel elements. **(E)** Acridine orange staining of a mistletoe plant (longitudinal) and its host (transverse). The white arrows mark the interface between the two species. Vessel elements (v), host wood fibres (f), and mistletoe parenchyma (p) are visible. **(F)** Toluidine blue staining of the interface between the main haustorium of the mistletoe (transverse) and its host (tangential). The black arrow marks mistletoe parenchyma cells with thickened cell walls. **(G,H)** Toluidine blue staining of the host-mistletoe interface. The host is oriented tangentially. The mistletoe haustorium is cut transversally. **(G)** Host vessel elements change their orientation and align with the mistletoe interface. **(H)** Host vessel elements are clustered in a swirl.

**FIGURE 8:**
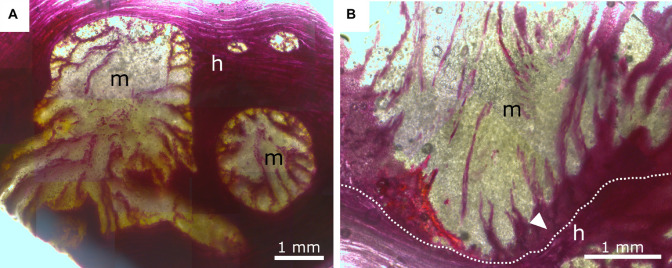
Phloroglucinol-HCl staining of mistletoe sinkers (m) and host wood (h). Red indicates tissue lignification. **(A)** Transverse plane of mistletoe sinkers, tangential plane of host wood. **(B)** Longitudinal plane of mistletoe haustorium, tangential plane of host wood. The dotted line marks the interface between the mistletoe and its host; the white arrow marks lignified mistletoe parenchyma.

To examine the anatomy of the mistletoe-host interface at the cellular and subcellular levels, we analyzed small blocks from the host wood containing sinkers of the mistletoe. The various tissues and their cross-sectional arrangement could be readily distinguished in thin sections highlighted with the selected stains. The lignified cell walls of mistletoe vessel elements, thick-walled parenchyma cells, and host wood fluoresce orange and yellow after being stained with acridine orange (ACO), deep blue after being stained with toluidine blue O (TBO), and red after being stained with phloroglucinol (PHO). Non-lignified thin-walled haustorial parenchyma cells were stained red with ACO, appeared purple with TBO, and remained unstained with PHO (Figures 7, 8).

In Figures 7A–H, the interface between the tissues of the mistletoe and the host wood can be clearly seen, regardless of the selected detail or magnification. The sinkers are wedge-shaped in longitudinal direction (Figure 7A). Bands of mistletoe vessels run along the wedge axis toward the tip and curve laterally toward the host tissue (Figures 7A,B). Close to the mistletoe haustorium, the vessel elements and fibers of the host wood change their direction (Figure 7G), form swirls or clusters (Figure 7H), and align with the mistletoe interface (Figures 7C,E).

The xylem of *A. flava* shows vessel elements with a diameter of 30–50 μm, pits, and open ends with grids that connect to the next element. Single or grouped vessel elements are surrounded by wood fibers with thickened cell walls (Figure 7C) and tapering ends. Lignified wood rays run perpendicular to wood fibers and vessel elements. Host wood is completely lignified, as has been verified by staining with ACO (Figures 7A–E) and PHO (Figure 8). Mistletoe sinkers possess slightly elongated vessel elements with diameters of approximately 40 μm embedded in isodiametric parenchyma cells having a diameter of approximately 25 μm (Figure 7E). Parenchyma cells possess thin walls in young sinkers, but they are thick-walled, especially when close to the mistletoe-host interface, in older sinkers (Figure 7F). The vessel bands split up into several bands near to the host interface (Figure 8A) and form clusters (Figure 7E). The resolution in Figures 7C,E,F allows individual host and mistletoe vessel elements to be distinguished. Details in Figures 7C,D show regions of direct contact of the water-conducting tissues of the two species, i.e., points at which the exchange of water and solutes is possible through the pits of the tracheids and vessels.

## Discussion

Like all mistletoe species, *V. album* benefits from the connection to its host by drawing water and solutes from the host vascular system (Heide-Jørgensen, 2008). Furthermore, its exposed location on host branches provides the epiphyte better access to sunlight. The multifunctional connection between the two species is not only subjected to mechanical loads, e.g., from wind, snow, animals, and the dead weight of the parasite, but must also grow in parallel with the annual growth of the host in order to maintain the continuous supply of water and nutrients to the mistletoe. The experiments conducted in this study provide insights into the morphology of the mistletoe endophyte, the anatomy of the interface with its host, and the dependence of the interface on the age and sex of the plant.

### Sex, Age, and Morphometrics of Mistletoe

Of all harvested samples, 11.5% were non-flowering juveniles, i.e., so young that their age could not be determined beyond doubt. This number was within the range of 8.2–13.4% reported by Stanton et al. (2009) for *V. album* in two habitats in Belgium. We found a female bias of 76.5% over all adult samples of the subspecies *V. album* ssp. *album* grown on *A. flava*, which was moderately higher than the values of 57 and 61% found in Belgium and just within the range recorded for European collection sites by Barlow et al. (1978) of between 72.9% (from Luxembourg) and 76.5% (from England) for European collection sites (the latter study also reported values of 67.3% for California and 80.3% for Japan). For *V. album* grown from seed, a female proportion of 67.3% was found (Showler, 1974). Wiens et al. (1996) attributed this female bias in *Viscum*, which occurs independently of the host, to genetic factors. Environmental influences seem to play a minor role, if any.

As mistletoe ages with exponential branch growth (Grundmann et al., 2014), its water demand increases and with it the required contact area with the host. This can be achieved in young stages via several small, elongated sinkers, which also contribute to the mistletoe’s increasing need for anchorage in the host. These requirements are also reflected in the studied variables that show the strongest linear correlation with age: the attachment diameter of the mistletoe (*d*_*mist*_) and the size of the hypertrophic region (as the product of its length and diameter). The host branch diameters positively correlating with age can be explained by the regular growth of the host. Although age has a positive effect on the thickness and the length of the hypertrophic region, the negative correlation with $\frac{d_{max}}{l_{hyp}}$ indicates that the hypertrophy found in host branches infested with younger stages of the mistletoe occurs in a more localized manner, whereas with increasing epiphyte age, the expansion along the branch axis accounts for an increasingly larger proportion of growth. This assumption is also supported by the negative influence of the sex of adult plants, compared with the juveniles, on $\frac{d_{max}}{l_{hyp}}$. For adult mistletoe, an effect of sex has only been found for one variable of hypertrophy growth $\left(\frac{d_{max}}{d_{acro}}\right)$, suggesting that sex markedly influences the exophyte (Zuber, 2004) but has little, if any, influence on the shape of the endophyte.

### Morphology of the Mistletoe Endophyte

Over the last few decades, the development and morphology of the endophytic structures of *V. album* have repeatedly been an integral part of botanical research, especially of the sinkers and cortical strands (Thoday, 1951; Sallé, 1978; Smith and Gledhill, 1983; Heide-Jørgensen, 2008). However, earlier studies of the endophyte are confined to sample cuttings and observations by eye or microscopy. In contrast, microCT scans provide 3D representation of the location of endophytic mistletoe structures inside the host. Teixeira-Costa and Ceccantini (2016) have applied this method in order to visualize the endophyte of two mistletoe species of the genus *Phoradendron*, native to tropical regions of America. *Phoradendron* species form a holdfast, cortical strands that run along the host branch axis, and sinkers (Calvin, 1967). Like *V. album*, *Phoradendron* possesses a xylem–xylem connection via water-conducting vessels that run along the sinkers (Teixeira-Costa and Ceccantini, 2016). However, *V. album* seems to grow fewer sinkers and spread further along the host branch axis compared with *Pharadendron* (Figures 5, 6).

The sinker grew from the edge toward the center of the host branch and were connected to cortical strands that ran basipetally and acropetally along the host branch axis (Figure 6). The development of a prominent endophyte and the lack of secondary haustoria is typical for mistletoes of the family Visceae (Heide-Jørgensen, 2008). The spreading of the endophyte along the host varies for the seven genera of the family. *Phoradendron* forms comparatively short cortical strands, which can nevertheless reach lengths of up to 13 cm. *Viscum* is known as having an extremely extensive endophyte and lies only second to the genus *Arceuthobium* in this regard (Heide-Jørgensen, 2008). In our study, a mistletoe plant only 4 years old showed endophytic structures growing inside the host over a distance of more than 5 cm with several thin and proportionally large sinkers (Figure 5). This very early spread of the mistletoe in the host branch supports the recommendations to minimize the mistletoe population (if mistletoe containment is desired) by cutting the host branch preferably at a young mistletoe stage and generously around the attachment site in order to remove the entire endophyte and prevent new shoots. In samples of older mistletoes, one main sinker was more prominent and noticeably larger than the other sinkers (Figure 6). This suggests that the mistletoe forms an extensive endophyte with a large number of small sinkers at a young age in order to achieve a quick and sufficient water acquisition, with added mechanical support being provided by an increased haustorium at older stages. This probably serves to resist the forces generated by host wood growth [induced through hormonal manipulation of host cell differentiation by the mistletoe (Aloni, 2015; Hu et al., 2017; Figures 7G,H)] and to avoid the collapse of mistletoe structures. Most of the water in *Aesculus* is conducted within its more recently produced diffuse-porous wood. The wedge-shaped thickening of the mistletoe haustorium ensures the continuous growth of the contact area and thus of the continuous potential uptake of water. Thus, the expansion of the haustorium along the host branch (see Figures 6A,C,E) might also help to facilitate the increased demand for water supplies by increasing the mistletoe-host interface. Smith and Gledhill (1983) have suggested that the increased growth of the host wood, evident as hypertrophied wood tissue around and especially basal to the infection site, not only increases the ability of the parasite to absorb water, but also strengthens its mechanical anchorage.

### Anatomy of the Mistletoe-Host Interface

Because most anatomical studies have focused on the formation of the young haustorium of *V. album* (Thoday, 1951; Sallé, 1978), little knowledge is available about the anatomy of the developed haustorium and its interface with the host at a (sub-)cellular level. Smith and Gledhill (1983) have provided detailed insights into the connection between mistletoe and host and describe the way that bands of vessels run from the mistletoe meristem at the branch basis toward the tip of the haustorium and bend sideward connecting with the host interface. Similar results have been found in our anatomical studies (Figures 7A,B). The vessel elements are embedded in the sinker parenchyma and form a direct connection to the host vessel elements at the mistletoe-host interface (Figures 7C,D). No breakdown of the cell walls between the host vessels and the mistletoe tracheids, as described in literature (Smith and Gledhill, 1983), has been observed in this study. However, the host and mistletoe cells lie closely adjacent to each other, and pits on both sides indicate a direct connection between the vessels of the two species. Host wood occasionally shows changes in its growth behavior around the mistletoe (Figures 7G,H). Vessel elements and wood fibers are reoriented and aligned with the interface providing an enlarged connecting surface for water uptake by the mistletoe tracheids. This effect is often enhanced by an accumulation of tracheid cells near the interface. Whereas the vessel elements are always lignified, lignification of the haustorial parenchyma is mostly found in older haustoria (Smith and Gledhill, 1983). This process starts at the interface of the sinker and continues toward the center. A gradient of lignification is formed together with the bands of vessels, which split up close to the host interface (Figure 8B). Such structural and chemical gradients might prevent stress peaks at the interface and, thus, guarantee structural integrity.

### Advances on the 3D Reconstruction of Haustoria

Masumoto et al. (2021) have used stacks of cell-type-labeled microscopic images to create a 3D representation of the haustoria of the obligate parasite *Striga hermonthica* and the facultative parasite *Phtheirospermum japonicum*. The advantage of this method is that it can distinguish between individual tissues of the haustorium, based on the cellular segmentation of the layers. However, the method is accompanied with an increased time requirement and is more applicable for haustoria up to 1 mm and not for haustoria up to several centimeters like those of *V. album*.

In our study, the sinkers of the mistletoe showed sufficient contrast against host wood (Figure 6). However, little to no contrast was apparent between the cortical strands of the mistletoe and the host bark. Teixeira-Costa and Ceccantini (2016) applied two different contrast agents (namely Lugol‘s solution and lead nitrate solution) to overcome such obstacles, resulting in an improvement in the contrast of the cortical strands, but also in a blurring of any contrast with the surrounding tissues. Thus, multiple scans of differently prepared samples had to be conducted to adequately visualize all tissues. Since cortical strands appear green because of their chlorophyll content, they are directly distinguishable from the surrounding brown host tissue by the naked eye (Figure 4; Heide-Jørgensen, 2008). Our approach of sectioning the samples after the microCT scan and taking images by stereo microscopy, therefore, offers the possibility of a manual segmentation of all mistletoe tissues from a single sample (Figures 4, 5). The approach of combining modern imaging methods with classical light microscopy, also offers the advantage of generating a 3D overview of the entire endophyte and a detailed examination of the selected layers.

The segmentation of the gray values of microCT data sets is increasingly being automated by algorithms due to the advancing technical possibilities (Théroux-Rancourt et al., 2020). However, our study demonstrated that especially when several tissues with (almost) the same gray level are located close to each other, a in-depth biological knowledge and a considerable amount of time are required for a precise segmentation (or, in the best case, to verify the correctness of the segmentation after automated segmentation). In addition, depending on the scientific question, it must be clarified whether the resolution and abstraction of the data sets associated with the scanning and the segmentation of the sample has a valid significance for that task.

## Conclusion

Based on morphometric and morphological (microCT) analyses, this study demonstrates that age has an effect on both the external and the internal appearance of the connection between the European mistletoe and its host. Even extremely young mistletoe plants show a large number of equivalent sinkers, with the endophyte extending over more than 5 cm within the host. Indeed, the resulting hypertrophy thickens the host branch by about 50%. Although this hypertrophic ratio changes only little with increasing mistletoe age, hypertrophy extends further acropetally and basipetally along the branch. Our methodological approach allows the creation of a 3D geometry of an entire mistletoe endophyte by merging several microCT scans and performing a segmentation of the tissues based on corresponding binocular images. The internal structures reveal that the individual sinkers in the attachment zone progressively merge and form a coherent structure in the older mistletoe plants.

Statistical analyses of the 130 mistletoes examined here have shown that their sex has no or little influence on the morphometry of the attachment region. However, the female bias was very pronounced at over 76%. The anatomical images of the mistletoe-host interface demonstrate, in addition to the directly adjacent conducting elements of both species, a gradual lignification of the mistletoe parenchyma in this particular region. Mistletoe-induced abnormal wood growth of the host (e.g., alignment, swirls) indicates the growth-induced competition for space and water and the mechanical stresses that occur between the two species at the cellular and tissue levels. In response to the permanently changing growth-dependent relationship of the host and the parasite, a dynamic spatiotemporal mechanical equilibrium is established in the attachment zone.

## Data Availability Statement

The original contributions presented in the study are included in the article/Supplementary Material, further inquiries can be directed to the corresponding author.

## Author Contributions

TS and OS acquired the funding for this study. MM, OS, and TS planned, designed, and supervised the research. MH produced the light microscopic images and recorded the morphometric data. OS and MM analyzed the morphometric data, performed the statistics, wrote the first draft of the manuscript and discussed, together with TS, the interpretation of the data. MH, MM, and AD obtained the microCT recordings and carried out the reconstructions and visualizations. RS and FW helped with the microCT recordings and their evaluation. All authors contributed significantly to the final version of the manuscript and gave their final approval.

## Conflict of Interest

The authors declare that the research was conducted in the absence of any commercial or financial relationships that could be construed as a potential conflict of interest.

## Publisher’s Note

All claims expressed in this article are solely those of the authors and do not necessarily represent those of their affiliated organizations, or those of the publisher, the editors and the reviewers. Any product that may be evaluated in this article, or claim that may be made by its manufacturer, is not guaranteed or endorsed by the publisher.

## Supplementary Material

Supplementary Table 1 Raw data of diameters and lengths of host branch (hypertrophy) and mistletoe.

Supplementary Table 2 Sample information and scan and segmentation settings for microCT.

Supplementary Table 3 Statistics of diameters and lengths of host branch and mistletoe with respect to normal distribution, homoscedasticity, and dependency of age and sex.

Supplementary Video 1 Video of reconstructed microCT recordings of the 4-year-old mistletoe *Viscum album* ssp. *album* in transverse sections from apical to basal.

Supplementary Video 2 Video of reconstructed microCT recordings of the 8-year-old mistletoe *Viscum album* ssp. *album* in transverse sections from apical to basal.

Supplementary Video 3 Video of reconstructed microCT recordings of the 17-year-old mistletoe *Viscum album* ssp. *album* in transverse sections from apical to basal.

Supplementary Video 4 Three-dimensional visualization of a segmented microCT scan of the host tree *Aesculus flava* and the 4-year-old mistletoe *Viscum album* ssp. *album*. The haustorial system of the mistletoe is highlighted in green, the outer hull of the host branch is colored in transparent brown.
