# Supplementary material for: Advances on the Visualization of the Internal Structures of the European Mistletoe: 3D Reconstruction Using Microtomography
Source: Front Plant Sci. 2021 Sep 20;12:715711. doi: 10.3389/fpls.2021.715711 (PMC8488221; doi:10.3389/fpls.2021.715711)
Supplement: Supplementary Table 1 — Raw data of diameters and lengths of host branch (hypertrophy) and mistletoe. [file Data_Sheet_1.PDF]

Sample	sex	age [a]	d_max/ l_hyp []	d_max * l_hyp [mm²]	d_max/((d_basi+d_acro)/2) []	d_max/ d_basi []	d_max/ d_acro []	mean d_max [mm]	mean d_acro [mm]	mean d_basi [mm]	mean d_mist [mm]	mean l_hyp [mm]
1	female	6	4.45	165.52	1.42	1.47	1.37	27.14	19.74	18.43	10.95	6.10
2	female	6	4.04	233.24	1.34	1.09	1.75	30.69	17.56	28.13	8.13	7.60
3	female	6	2.22	532.50	1.42	1.60	1.27	34.36	27.03	21.52	22.66	15.50
4	female	7	4.06	580.18			1.46	48.51	33.34			11.96
5	female	7	2.11	279.00	1.42	1.41	1.42	24.24	17.11	17.14		11.51
6	female	7	1.95	1268.63	1.18	1.20	1.16	49.75	42.80	41.60	9.72	25.50
7	female	7	2.44	323.27	1.59	1.61	1.56	28.11	18.00	17.47	9.05	11.50
8	female	7	3.47	382.10	1.62	1.50	1.76	36.39	20.70	24.19	13.60	10.50
9	female	7	4.91	182.57	1.44	1.65	1.29	29.93	23.29	18.15	13.04	6.10
10	female	7	4.38	227.12	1.22	1.24	1.20	31.55	26.29	25.52	13.25	7.20
11	female	7	3.77	456.50	1.09	1.11	1.07	41.50	38.69	37.37	12.03	11.00
12	female	7	3.39	292.95	1.09	1.06	1.13	31.50	27.93	29.80	8.64	9.30
13	female	7	2.21	974.93	1.49	1.67	1.35	46.43	34.51	27.86	14.61	21.00
14	female	7	3.95	142.29	1.60	1.60	1.59	23.72	14.91	14.80	17.21	6.00
15	female	8	4.42	178.59	2.45	2.19	2.78	28.08	10.09	12.83		6.36
16	female	8	2.22	399.08	1.58	1.33	1.93	29.76	15.42	22.30		13.41
17	female	8	2.48	357.12	1.07	1.07	1.07	29.76	27.82	27.84		12.00
18	female	8	3.42	768.98	1.05	0.99	1.12	51.27	45.70	52.04		15.00
19	female	8	5.21	654.08	1.28	1.42	1.17	58.40	50.10	41.05		11.20
20	female	8	3.47	382.78	1.09	1.18	1.01	36.46	36.08	30.96		10.50
21	female	8	1.56	1208.47	1.25	1.25	1.25	43.47	34.71	34.87	8.43	27.80
22	female	8	3.25	293.50	1.94	2.11	1.79	30.90	17.27	14.64	20.26	9.50
23	female	8	3.58	258.83	1.30	1.31	1.29	30.45	23.52	23.30	14.07	8.50
24	female	8	3.15	395.53	1.37	1.52	1.26	35.32	28.12	23.28	14.00	11.20
25	female	8	2.97	303.15	1.48	1.68	1.32	30.02	22.73	17.87	22.99	10.10
26	female	8	3.78	378.10	1.35	1.37	1.33	37.81	28.43	27.66	13.26	10.00
27	female	8	3.81	329.27	1.36	1.47	1.26	35.41	28.04	24.09	10.86	9.30
28	female	8	3.13	221.09	1.40	1.47	1.34	26.32	19.57	17.92	12.97	8.40
29	female	8	2.60	569.87	2.34	2.45	2.23	38.51	17.24	15.73	27.56	14.80
30	female	8	4.19	160.92	1.43	1.49	1.38	25.96	18.84	17.39	15.16	6.20
31	female	8	2.78	225.50		1.98		25.06		12.66	11.00	9.00
32	female	8	3.72	238.00	1.41	1.37	1.45	29.75	20.50	21.72	11.28	8.00
33	female	8	1.90	399.55	1.71	1.70	1.73	27.56	15.97	16.25	18.95	14.50
34	female	8	2.99	322.87	1.59	1.79	1.43	31.05	21.68	17.32	28.11	10.40
35	female	8	2.09	213.62	1.28	1.38	1.19	21.15	17.77	15.32	25.68	10.10
36	female	8	3.62	231.88	2.22	2.27	2.17	28.99	13.36	12.80	22.89	8.00
37	female	9	5.14	309.47			1.25	39.88	31.82			7.76
38	female	9	2.65	1339.43	1.37	1.32	1.42	59.53	41.80	45.16		22.50

Sample	sex	age [a]	d_max/ l_hyp []	d_max * l_hyp [mm²]	d_max/((d_basi+d_acro)/2) []	d_max/ d_basi []	d_max/ d_acro []	mean d_max [mm]	mean d_acro [mm]	mean d_basi [mm]	mean d_mist [mm]	mean l_hyp [mm]
39	female	9			0.98	0.95	1.00	42.79	42.77	44.92	17.83	
40	female	9			1.02	0.94	1.11	46.96	42.13	50.06	13.34	
41	female	9	2.53	1226.72	1.25	1.42	1.12	55.76	49.78	39.35	12.36	22.00
42	female	9	3.19	294.43	1.32	1.40	1.25	30.67	24.52	21.90	15.05	9.60
43	female	9	3.10	297.68	2.60	2.49	2.71	30.38	11.23	12.18	29.54	9.80
44	female	9	4.32	283.26	1.50	1.81	1.28	34.97	27.43	19.31	32.57	8.10
45	female	9	3.92	175.84	1.21	1.16	1.27	26.25	20.59	22.71	18.30	6.70
46	female	9	3.29	556.73	1.32	1.30	1.35	42.83	31.79	32.97	18.61	13.00
47	female	9	7.05	213.40	1.34	1.16	1.60	38.80	24.32	33.57	17.60	5.50
48	female	9	1.91	1717.20	1.31	1.26	1.35	57.24	42.41	45.28	20.01	30.00
49	female	9	3.56	423.08	1.82	1.78	1.86	38.82	20.85	21.83	20.88	10.90
50	female	9									18.00	
51	female	9	3.85	601.00	1.70	1.73	1.67	48.08	28.83	27.72	34.00	12.50
52	female	10	3.21	375.89	1.67	1.75	1.60	34.74	21.67	19.82		10.82
53	female	10	2.00	2314.89	1.44	1.26	1.68	68.09	40.51	53.97	12.72	34.00
54	female	10	3.42	551.69	1.16	1.07	1.27	43.44	34.29	40.67	28.74	12.70
55	female	10	4.47	394.71	1.73	1.67	1.80	41.99	23.32	25.13	37.71	9.40
56	female	10	3.05	554.99	1.21	1.10	1.35	41.11	30.38	37.51	20.91	13.50
57	female	11	4.38	448.11	1.39	1.25	1.57	44.28	28.13	35.50		10.12
58	female	11	3.33	781.78	2.16	2.71	1.80	51.03	28.35	18.86		15.32
59	female	11	3.25	467.64	3.42	3.28	3.56	38.97	10.95	11.87	33.66	12.00
60	female	11			0.93	0.93	0.92	38.01	41.12	40.80	10.49	
61	female	11	2.00	2314.89	1.44	1.26	1.68	68.09	40.51	53.97	14.37	34.00
62	female	11	2.39	574.04	1.52	1.20	2.05	37.04	18.06	30.84	24.53	15.50
63	female	11	5.14	493.48	2.03	2.14	1.93	50.36	26.12	23.57	56.10	9.80
64	female	11	2.46	1085.39	2.04	1.82	2.34	51.69	22.13	28.43	56.85	21.00
65	female	12	2.36	924.26	1.51	1.39	1.65	46.68	28.30	33.63		19.80
66	female	12	2.24	608.77	1.31	1.41	1.22	36.90	30.29	26.10	31.91	16.50
67	female	12	2.29	1795.50	1.77	2.04	1.56	64.13	41.07	31.42	27.44	28.00
68	female	12	2.29	1795.50	1.77	2.04	1.56	64.13	41.07	31.42	25.39	28.00
69	female	12	1.81	876.59	2.21	2.01	2.44	39.85	16.30	19.84	34.89	22.00
70	female	12	3.09	894.37	1.94	2.01	1.87	52.61	28.17	26.14	38.69	17.00
71	female	12	2.13	2608.38	1.59	1.49	1.70	74.53	43.77	50.17	41.31	35.00
72	female	13			1.25	1.24	1.26	52.79	41.79	42.47		
73	female	13	4.06	430.90	1.13	1.05	1.23	41.84	34.10	39.69		10.30
74	female	13	5.60	215.45	1.25	1.20	1.31	34.75	26.59	28.97	16.40	6.20
75	female	14			1.25	1.24	1.26	52.79	41.79	42.47		
76	female	14	2.71	1115.69	2.44	2.11	2.88	54.96	19.11	26.00		20.30

Sample	sex	age [a]	d_max/ l_hyp []	d_max * l_hyp [mm²]	d_max/((d_basi+d_acro)/2) []	d_max/ d_basi []	d_max/ d_acro []	mean d_max [mm]	mean d_acro [mm]	mean d_basi [mm]	mean d_mist [mm]	mean l_hyp [mm]
77	female	14	2.89	416.10	1.55	1.73	1.41	34.68	24.52	20.09	35.15	12.00
78	female	14	2.72	882.09	1.78	1.79	1.77	49.01	27.74	27.35	32.71	18.00
79	female	14	3.29	556.73	1.32	1.30	1.35	42.83	31.79	32.97	36.73	13.00
80	female	14	2.00	1106.73	1.32	1.51	1.18	47.10	39.91	31.19	31.57	23.50
81	female	14	2.35	1088.33	1.58	1.58	1.57	50.62	32.16	31.98	33.03	21.50
82	female	15	2.74	887.85	1.79	1.59	2.05	49.33	24.09	31.11	34.31	18.00
83	female	16	3.03	511.55	1.19	1.04	1.38	39.35	28.47	37.86	35.02	13.00
84	female	17	4.82	741.46	1.39	1.23	1.59	59.80	37.56	48.51	51.68	12.40
85	female	17	1.93	1459.84	1.43	1.38	1.48	53.09	35.96	38.39	52.64	27.50
86	female	18	1.99	1647.50	1.54	1.36	1.77	57.21	32.29	42.19		28.80
87	female	18	2.36	2411.68	1.90	1.83	1.98	75.37	38.15	41.13	53.16	32.00
88	female	19	2.45	1719.85	1.53	1.73	1.37	64.90	47.38	37.61		26.50
89	male	6	2.60	1374.14	1.34	1.27	1.41	59.75	42.24	46.90		23.00
90	male	6	2.63	317.68	1.22	1.08	1.40	28.88	20.66	26.65	10.77	11.00
91	male	6	4.90	142.88	1.37	1.20	1.59	26.46	16.63	22.10	8.12	5.40
92	male	6	3.77	241.24	1.46	1.39	1.53	30.16	19.65	21.62	11.96	8.00
93	male	7	1.98	262.49	1.33	1.33	1.33	22.81	17.11	17.14		11.51
94	male	7	2.79	670.84	1.77	1.40	2.40	43.28	18.06	30.84	14.85	15.50
95	male	7	2.62	1048.00	2.38	2.31	2.44	52.40	21.47	22.66	44.77	20.00
96	male	7	5.63	388.19	1.43	1.35	1.51	46.77	30.93	34.62	13.55	8.30
97	male	7	4.41	63.61	1.34	1.16	1.59	16.74	10.50	14.41	8.01	3.80
98	male	7	3.79	122.98	1.59	1.59	1.59	21.58	13.58	13.54	12.22	5.70
99	male	8	3.83	697.21	1.33	1.34	1.33	51.65	38.86	38.56	14.50	13.50
100	male	8	2.96	267.05	1.79	1.63	1.99	28.11	14.12	17.23	11.75	9.50
101	male	8	2.55	167.10	1.57	1.49	1.66	20.63	12.45	13.87	14.18	8.10
102	male	8	3.82	359.14	2.34	2.37	2.31	37.03	16.04	15.62	15.59	9.70
103	male	8	4.15	272.12	2.09	1.88	2.37	33.60	14.18	17.91	17.64	8.10
104	male	8	4.32	242.74	1.93	1.92	1.94	32.37	16.65	16.89	16.80	7.50
105	male	8					1.61	36.09	22.48		15.78	
106	male	9	3.16	303.85	1.52	1.62	1.43	31.01	21.62	19.19	14.07	9.80
107	male	10	2.87	674.39	1.66	1.55	1.78	44.02	24.70	28.35		15.32
108	male	10	3.39	563.99	1.41	1.22	1.68	43.72	26.06	35.86	17.70	12.90
109	male	10	1.86	476.48	2.46	2.56	2.36	29.78	12.61	11.66	16.07	16.00
110	male	12	2.60	1374.14	1.34	1.27	1.41	59.75	42.24	46.90		23.00
111	male	12	3.42	986.94	1.18	1.21	1.15	58.06	50.49	48.07	19.78	17.00
112	male	13	2.20	754.52	1.51	1.40	1.64	40.79	24.88	29.16	25.13	18.50
113	male	14	2.66	681.84	1.58	2.02	1.30	42.62	32.77	21.11	34.88	16.00
114	male	14	2.12	2304.39	1.66	1.68	1.65	69.83	42.40	41.53	24.40	33.00

Sample	sex	age [a]	d_max/ l_hyp []	d_max * l_hyp [mm^2]	d_max/((d_basi+d_acro)/2) []	d_max/ d_basi []	d_max/ d_acro []	mean d_max [mm]	mean d_acro [mm]	mean d_basi [mm]	mean d_mist [mm]	mean l_hyp [mm]
115	male	21	2.50	2406.22	2.26	2.17	2.36	77.62	32.89	35.79		31.00
116	juvenile	3	6.79	78.47	1.11	1.08	1.15	23.08	20.08	21.42	3.19	3.40
117	juvenile	3	6.30	64.56	1.19	1.19	1.18	20.18	17.03	16.90	2.67	3.20
118	juvenile	3	3.54	20.40	1.57	1.48	1.66	8.50	5.11	5.73	3.22	2.40
119	juvenile	3	8.62	41.73	1.12	1.15	1.09	18.97	17.34	16.56	3.77	2.20
120	juvenile	3	3.84	92.32	1.21	1.17	1.26	18.84	15.00	16.16	4.33	4.90
121	juvenile	3	7.00	25.26	1.08	1.04	1.14	13.30	11.69	12.82	5.38	1.90
122	juvenile	4	3.34	163.52	1.27	1.30	1.25	23.36	18.70	17.96	3.99	7.00
123	juvenile	4	6.37	46.45	1.08	1.10	1.06	17.21	16.26	15.63	3.76	2.70
124	juvenile	4	8.54	45.16	1.17	1.10	1.25	19.64	15.69	17.88	3.59	2.30
125	juvenile	5			1.04	1.04	1.04	37.12	35.67	35.86		
126	juvenile	5	5.70	110.40	1.13	1.22	1.04	25.09	24.02	20.50	5.17	4.40
127	juvenile	5	3.71	71.85	1.47	1.43	1.51	16.33	10.79	11.38	6.64	4.40
128	juvenile	6	4.69	163.34	1.57	1.46	1.68	27.69	16.45	18.91	9.45	5.90
129	juvenile	6	3.01	232.98	1.33	1.24	1.43	26.48	18.57	21.28	7.21	8.80
130	juvenile	6	3.31	153.17	1.30	1.23	1.38	22.53	16.33	18.34	10.52	6.80
**Mean**			3.45	618.82	1.52	1.51	1.56	39.22	26.47	27.25	19.82	13.38
**SD**			1.33	580.06	0.39	0.41	0.43	14.13	10.41	11.13	12.50	7.86
**Median**			**3.23**	**397.30**	**1.42**	**1.40**	**1.43**	**37.12**	**24.61**	**25.32**	**15.92**	**11.10**
**IQR**			**1.46**	**545.53**	**0.38**	**0.47**	**0.45**	**20.02**	**16.43**	**17.80**	**16.26**	**8.85**
**Max**			**8.62**	**2608.38**	**3.42**	**3.28**	**3.56**	**77.62**	**50.49**	**53.97**	**56.85**	**35.00**
**Min**			**1.56**	**20.40**	**0.93**	**0.93**	**0.92**	**8.50**	**5.11**	**5.73**	**2.67**	**1.90**
n			**122**	**122**	**125**	**126**	**128**	**129**	**128**	**126**	**104**	**122**
**Median**		female	3.12	**462.07**	1.42	1.42	1.42	39.85	28.08	27.84	**20.13**	12.00
**IQR**		female	1.44	**597.50**	0.40	0.49	0.48	19.52	15.31	17.77	**19.29**	9.83
**Median**		male	2.92	**432.34**	1.54	1.45	1.61	37.03	21.47	22.38	**15.22**	12.21
**IQR**		male	1.21	**476.57**	0.44	0.54	0.54	19.88	15.51	18.33	**5.13**	8.60
**Median**		juvenile	5.20	**75.16**	1.19	1.19	1.25	20.18	16.45	17.88	**4.16**	3.90
**IQR**		juvenile	3.10	**96.99**	0.20	0.17	0.29	6.20	3.29	3.81	**2.69**	3.18
