# Supplementary material for: Advances on the Visualization of the Internal Structures of the European Mistletoe: 3D Reconstruction Using Microtomography
Source: Front Plant Sci. 2021 Sep 20;12:715711. doi: 10.3389/fpls.2021.715711 (PMC8488221; doi:10.3389/fpls.2021.715711)
Supplement: Supplementary Table 2 — Sample information and scan and segmentation settings for microCT. [file Data_Sheet_2.PDF]

X-ray microtomography samples and related scanning and reconstruction settings

Sample		Scanning parameters												Reconstruction parameters				
Age [years]	Sex	Scanner	Software	Resolution [µm]	Source voltage [kV]	Source current [µA]	360° scan	Batch scan	Rotation step [°]	Filter	Frame averaging	Random movement correction	Scan duration	Software	Total slices	Ring artefact correction	Beam hardening correction [%]	Smoothing
4	juvenile	Bruker SkyScan 1272	SkyScan 1.1.10	10	50	200	yes	yes	0.6	No Filter	3	10	3h 16 min	NRecon 1.6.10.1	5402	18	20	0
8	female	Nikon XT H 160	Inspect X, XT 4.4.2	86	130	62	yes	no	0.23	No Filter	8	---	1h 59 min	CT-Pro 3D, XT 4.4.2	1008	yes	0	0
17	female	Nikon XT H 160	Inspect X, XT 4.4.3	86	130	62	yes	no	0.23	No Filter	8	---	1h 49 min	CT-Pro 3D, XT 4.4.3	1008	yes	0	0
