# Supplementary material for: Advances on the Visualization of the Internal Structures of the European Mistletoe: 3D Reconstruction Using Microtomography
Source: Front Plant Sci. 2021 Sep 20;12:715711. doi: 10.3389/fpls.2021.715711 (PMC8488221; doi:10.3389/fpls.2021.715711)
Supplement: Supplementary Table 3 — Statistics of diameters and lengths of host branch and mistletoe with respect to normal distribution, homoscedasticity, and dependency of age and sex. [file Data_Sheet_3.PDF]

```
> #######################################
> ### Statistics Mistletoe Morphology ###
> #######################################
>
> ##Date##
> date <- "22.04.2021 by Olga Speck & Max Mylo"
>
> library(stats)
> library(ggpubr)
> library(car)
> setwd("E:/# Publikationen/2021- Mistel Morphologie/Zusätzliche Dateien")
>
>
> #define your own numeric class
> setClass('myNum')
> #define conversion
> setAs("character","myNum", function(from) as.numeric(gsub(",","\\.",gsub("\\.","",from))))
> Mistletoe_Morphology=read.csv2("R_Mistletoe_Statistik_PK.csv", stringsAsFactors=FALSE,
+         colClasses=c("myNum","myNum","myNum","myNum","myNum","myNum","myNum","myNum","myNum","m
Warning messages:
1: In asMethod(object) : NAs introduced by coercion
2: In asMethod(object) : NAs introduced by coercion
>
> Mistletoe_MorphologyJuvenileFirst <- read.csv2(file= "R_Mistletoe_StatistikJuvenileFirst.csv",
+                                                header=TRUE, sep=";", dec=",", fill=TRUE)
> Mistletoe_MorphologyFemaleFirst <- read.csv2(file= "R_Mistletoe_StatistikFemaleFirst.csv",
+                                              header=TRUE, sep=";", dec=",", fill=TRUE)
>
>
> ###AGE###
> ## Shapiro-Test for normal distribution##
> ## Age [a] alle -->> not-normally distributed
> shapiro.test (Mistletoe_Morphology$Alter)

        Shapiro-Wilk normality test

data:  Mistletoe_Morphology$Alter
W = 0.94143, p-value = 2.637e-05

>
> ## d_max mean [mm] alle -->> not-normally distributed
> shapiro.test (Mistletoe_Morphology$d_max)

        Shapiro-Wilk normality test

data:  Mistletoe_Morphology$d_max
W = 0.97773, p-value = 0.03203

>
> ## d_acro mean [mm] alle -->> not-normally distributed
> shapiro.test (Mistletoe_Morphology$d_acro)

        Shapiro-Wilk normality test

data:  Mistletoe_Morphology$d_acro
W = 0.96051, p-value = 0.0008921

>
> ## d_basi mean [mm] alle -->> not-normally distributed
> shapiro.test (Mistletoe_Morphology$d_basi)

        Shapiro-Wilk normality test
```

```
data:  Mistletoe_Morphology$d_basi
W = 0.9516, p-value = 0.0001936

>
> ## d_mist [mm] alle -->> not-normally distributed
> shapiro.test (Mistletoe_Morphology$d_mist)

        Shapiro-Wilk normality test

data:  Mistletoe_Morphology$d_mist
W = 0.90783, p-value = 2.273e-06

>
> ## l_hyp [mm] alle -->> not-normally distributed
> shapiro.test (Mistletoe_Morphology$l_hyp)

        Shapiro-Wilk normality test

data:  Mistletoe_Morphology$l_hyp
W = 0.90904, p-value = 4.889e-07

>
> ## d_max/l_hyp [mm] alle -->> not-normally distributed
> shapiro.test (Mistletoe_Morphology$d_max.l_hyp)

        Shapiro-Wilk normality test

data:  Mistletoe_Morphology$d_max.l_hyp
W = 0.88099, p-value = 1.913e-08

>
> ## d_max*l_hyp [mm²] alle -->> not-normally distributed
> shapiro.test (as.numeric(Mistletoe_Morphology$d_max.l_hyp.1))

        Shapiro-Wilk normality test

data:  as.numeric(Mistletoe_Morphology$d_max.l_hyp.1)
W = 0.80307, p-value = 1.701e-11

>
> ## d_max/((d_basi+d_acro)/2) [/] alle -->> not-normally distributed
> shapiro.test (Mistletoe_Morphology$d_max.d_basi.d_acro)

        Shapiro-Wilk normality test

data:  Mistletoe_Morphology$d_max.d_basi.d_acro
W = 0.87207, p-value = 5.508e-09

>
> ## d_max/d_basi[/] alle -->> not-normally distributed
> shapiro.test (Mistletoe_Morphology$d_max.d_basi)

        Shapiro-Wilk normality test

data:  Mistletoe_Morphology$d_max.d_basi
W = 0.89925, p-value = 1.022e-07

>
> ## d_max/d_acro[/] alle -->> not-normally distributed
> shapiro.test (Mistletoe_Morphology$d_max.d_acro)
```

```
        Shapiro-Wilk normality test

data:  Mistletoe_Morphology$d_max.d_acro
W = 0.8737, p-value = 4.793e-09

>
>
> ###Levene Test####
> leveneTest(Mistletoe_Morphology$d_max, Mistletoe_Morphology$d_acro)
Levene's Test for Homogeneity of Variance (center = median)
       Df    F value    Pr(>F)
group 119 9.9592e+27 < 2.2e-16 ***
        8
---
Signif. codes:  0 '***' 0.001 '**' 0.01 '*' 0.05 '.' 0.1 ' ' 1
Warning messages:
1: In leveneTest.default(Mistletoe_Morphology$d_max, Mistletoe_Morphology$d_acro) :
  Mistletoe_Morphology$d_acro coerced to factor.
2: In anova.lm(lm(resp ~ group)) :
  ANOVA F-tests on an essentially perfect fit are unreliable
> leveneTest(Mistletoe_Morphology$d_max, Mistletoe_Morphology$d_basi)
Levene's Test for Homogeneity of Variance (center = median)
       Df    F value    Pr(>F)
group 117 7.4834e+28 < 2.2e-16 ***
        8
---
Signif. codes:  0 '***' 0.001 '**' 0.01 '*' 0.05 '.' 0.1 ' ' 1
Warning messages:
1: In leveneTest.default(Mistletoe_Morphology$d_max, Mistletoe_Morphology$d_basi) :
  Mistletoe_Morphology$d_basi coerced to factor.
2: In anova.lm(lm(resp ~ group)) :
  ANOVA F-tests on an essentially perfect fit are unreliable
> leveneTest(Mistletoe_Morphology$d_basi, Mistletoe_Morphology$d_acro)
Levene's Test for Homogeneity of Variance (center = median)
       Df    F value    Pr(>F)
group 116 2.4263e+28 < 2.2e-16 ***
        8
---
Signif. codes:  0 '***' 0.001 '**' 0.01 '*' 0.05 '.' 0.1 ' ' 1
Warning messages:
1: In leveneTest.default(Mistletoe_Morphology$d_basi, Mistletoe_Morphology$d_acro) :
  Mistletoe_Morphology$d_acro coerced to factor.
2: In anova.lm(lm(resp ~ group)) :
  ANOVA F-tests on an essentially perfect fit are unreliable
>
> ##############################
> # Friedmann-Test with post-hoc#
> R_Mistletoe_Statistik <- read.csv2(file= "R_Mistletoe_Statistik.csv",header=TRUE,
+                                     sep=";", dec=",", fill=TRUE)
> matrix <- as.matrix(R_Mistletoe_Statistik)
>
> friedman.test(diameter~localisation|probe, data=matrix)

        Friedman rank sum test

data:  diameter and localisation and probe
Friedman chi-squared = 174.74, df = 2, p-value < 2.2e-16

> compare_means(diameter~localisation, R_Mistletoe_Statistik,
+               method="wilcox.test",alternative = "two.sided",
+               paired=TRUE, p.adjust.methods="holm")
# A tibble: 3 x 8
```

```
  .y.      group1 group2        p   p.adj p.format p.signif method
  <chr>    <chr>  <chr>     <dbl>   <dbl> <chr>    <chr>    <chr>
1 diameter d_acro d_basi 2.69e- 2 2.7e- 2 0.027    *        Wilcoxon
2 diameter d_acro d_max  3.91e-22 1.2e-21 <2e-16   ****     Wilcoxon
3 diameter d_basi d_max  1.02e-21 2  e-21 <2e-16   ****     Wilcoxon
>
>
> ####Spearman & Pearson Correlation analyses with age########
> ### D max ###
> Spearman_dMax = cor.test(Mistletoe_Morphology$Alter, Mistletoe_Morphology$d_max,
 method = "spearman")        #### Rank correlation
Warning message:
In cor.test.default(Mistletoe_Morphology$Alter, Mistletoe_Morphology$d_max,  :
  Kann exakten p-Wert bei Bindungen nicht berechnen
> Spearman_dMax$estimate
      rho
0.6914435
>
> Pearson_dMax  = cor.test(Mistletoe_Morphology$Alter, Mistletoe_Morphology$d_max,
 method = "pearson")          #### Linear Correlation
> Pearson_dMax$estimate
      cor
0.7010392
> Pearson_dMax$estimate*Pearson_dMax$estimate
     cor
0.491456
>
> ### D acro ###
> Spearman_dAcro = cor.test(Mistletoe_Morphology$Alter, Mistletoe_Morphology$d_acro,
 method = "spearman")
Warning message:
In cor.test.default(Mistletoe_Morphology$Alter, Mistletoe_Morphology$d_acro,  :
  Kann exakten p-Wert bei Bindungen nicht berechnen
> Spearman_dAcro$estimate
      rho
0.4960559
>
> Pearson_dAcro  = cor.test(Mistletoe_Morphology$Alter, Mistletoe_Morphology$d_acro,
 method = "pearson")
> Pearson_dAcro$estimate
      cor
0.4663922
> Pearson_dAcro$estimate*Pearson_dAcro$estimate
      cor
0.2175217
>
> ### D basi ###
> Spearman_dBasi = cor.test(Mistletoe_Morphology$Alter, Mistletoe_Morphology$d_basi,
 method = "spearman")
Warning message:
In cor.test.default(Mistletoe_Morphology$Alter, Mistletoe_Morphology$d_basi,  :
  Kann exakten p-Wert bei Bindungen nicht berechnen
> Spearman_dBasi$estimate
      rho
0.5039506
>
> Pearson_dBasi  = cor.test(Mistletoe_Morphology$Alter, Mistletoe_Morphology$d_basi,
 method = "pearson")
> Pearson_dBasi$estimate
      cor
0.4813901
> Pearson_dBasi$estimate*Pearson_dBasi$estimate
```

```
      cor
0.2317364
>
> ### D mist ###
> Spearman_dMist = cor.test(Mistletoe_Morphology$Alter, Mistletoe_Morphology$d_mist,
 method = "spearman")
Warning message:
In cor.test.default(Mistletoe_Morphology$Alter, Mistletoe_Morphology$d_mist,  :
  Kann exakten p-Wert bei Bindungen nicht berechnen
> Spearman_dMist$estimate
      rho
0.8070118
>
> Pearson_dMist  = cor.test(Mistletoe_Morphology$Alter, Mistletoe_Morphology$d_mist,
 method = "pearson")
> Pearson_dMist$estimate
     cor
0.780944
> Pearson_dMist$estimate*Pearson_dMist$estimate
      cor
0.6098735
>
> ### L hyp ###
> Spearman_LHyp = cor.test(Mistletoe_Morphology$Alter, Mistletoe_Morphology$l_hyp,
 method = "spearman")
Warning message:
In cor.test.default(Mistletoe_Morphology$Alter, Mistletoe_Morphology$l_hyp,  :
  Kann exakten p-Wert bei Bindungen nicht berechnen
> Spearman_LHyp$estimate
      rho
0.6633592
>
> Pearson_LHyp  = cor.test(Mistletoe_Morphology$Alter, Mistletoe_Morphology$l_hyp,
 method = "pearson")
> Pearson_LHyp$estimate
      cor
0.6455243
> Pearson_LHyp$estimate*Pearson_LHyp$estimate
      cor
0.4167016
>
> ### D max * L hyp ###
> Spearman_DmaxLHyp = cor.test(Mistletoe_Morphology$Alter, Mistletoe_Morphology$d_max.l_hyp.1,
 method = "spearman")
Warning message:
In cor.test.default(Mistletoe_Morphology$Alter, Mistletoe_Morphology$d_max.l_hyp.1,  :
  Kann exakten p-Wert bei Bindungen nicht berechnen
> Spearman_DmaxLHyp$estimate
      rho
0.6984122
>
> Pearson_DmaxLHyp  = cor.test(Mistletoe_Morphology$Alter, Mistletoe_Morphology$d_max.l_hyp.1,
 method = "pearson")
> Pearson_DmaxLHyp$estimate
      cor
0.6353101
> Pearson_DmaxLHyp$estimate*Pearson_DmaxLHyp$estimate
      cor
0.4036189
>
> ### D max / L hyp ###
> Spearman_Dmax_LHyp = cor.test(Mistletoe_Morphology$Alter, Mistletoe_Morphology$d_max.l_hyp,
```

```
 method = "spearman")
Warning message:
In cor.test.default(Mistletoe_Morphology$Alter, Mistletoe_Morphology$d_max.l_hyp,  :
  Kann exakten p-Wert bei Bindungen nicht berechnen
> Spearman_Dmax_LHyp$estimate
       rho
-0.4129759
>
> Pearson_Dmax_LHyp  = cor.test(Mistletoe_Morphology$Alter, Mistletoe_Morphology$d_max.l_hyp,
 method = "pearson")
> Pearson_Dmax_LHyp$estimate
       cor
-0.4452346
> Pearson_Dmax_LHyp$estimate*Pearson_Dmax_LHyp$estimate
      cor
0.1982339
>
> ### D max / D acro ###
> Spearman_Dmax_DAcro = cor.test(Mistletoe_Morphology$Alter, Mistletoe_Morphology$d_max.d_acro,
 method = "spearman")
Warning message:
In cor.test.default(Mistletoe_Morphology$Alter, Mistletoe_Morphology$d_max.d_acro,  :
  Kann exakten p-Wert bei Bindungen nicht berechnen
> Spearman_Dmax_DAcro$estimate
      rho
0.2665412
>
> Pearson_Dmax_DAcro  = cor.test(Mistletoe_Morphology$Alter, Mistletoe_Morphology$d_max.d_acro,
 method = "pearson")
> Pearson_Dmax_DAcro$estimate
      cor
0.2405767
> Pearson_Dmax_DAcro$estimate*Pearson_Dmax_DAcro$estimate
       cor
0.05787717
>
> ### D max / D basi ###
> Spearman_Dmax_DBasi = cor.test(Mistletoe_Morphology$Alter, Mistletoe_Morphology$d_max.d_basi,
 method = "spearman")
Warning message:
In cor.test.default(Mistletoe_Morphology$Alter, Mistletoe_Morphology$d_max.d_basi,  :
  Kann exakten p-Wert bei Bindungen nicht berechnen
> Spearman_Dmax_DBasi$estimate
      rho
0.2641797
>
> Pearson_Dmax_DBasi  = cor.test(Mistletoe_Morphology$Alter, Mistletoe_Morphology$d_max.d_basi,
 method = "pearson")
> Pearson_Dmax_DBasi$estimate
      cor
0.2327968
> Pearson_Dmax_DBasi$estimate*Pearson_Dmax_DBasi$estimate
       cor
0.05419435
>
> ### D max / ((D basi + D acro) / 2) ###
> Spearman_Dmax_DBasi_DAcro = cor.test(Mistletoe_Morphology$Alter,
Mistletoe_Morphology$d_max.d_basi.d_acro, method = "spearman")
Warning message:
In cor.test.default(Mistletoe_Morphology$Alter, Mistletoe_Morphology$d_max.d_basi.d_acro,  :
  Kann exakten p-Wert bei Bindungen nicht berechnen
> Spearman_Dmax_DBasi_DAcro$estimate
```

```
      rho
0.2879521
>
> Pearson_Dmax_DBasi_DAcro  = cor.test(Mistletoe_Morphology$Alter,
Mistletoe_Morphology$d_max.d_basi.d_acro, method = "pearson")
> Pearson_Dmax_DBasi_DAcro$estimate
      cor
0.2492075
> Pearson_Dmax_DBasi_DAcro$estimate*Pearson_Dmax_DBasi_DAcro$estimate
       cor
0.06210439
>
>
>
> ######## Multiple Regression #########
> ### D max ###
> MultRegVSJuvenile_DMAx <- lm(d_max ~ Alter + Geschlecht,
data = Mistletoe_MorphologyJuvenileFirst)
> summary(MultRegVSJuvenile_DMAx)

Call:
lm(formula = d_max ~ Alter + Geschlecht, data = Mistletoe_MorphologyJuvenileFirst)

Residuals:
    Min      1Q  Median      3Q     Max
-18.016  -6.926  -1.064   4.962  27.688

Coefficients:
                     Estimate Std. Error t value Pr(>|t|)
(Intercept)           10.3587     2.9041   3.567 0.000513 ***
Alter                  2.5863     0.3034   8.523 4.24e-14 ***
GeschlechtB (female)   5.6264     3.3399   1.685 0.094568 .
GeschlechtC (male)     6.1852     3.5843   1.726 0.086884 .
---
Signif. codes:  0 '***' 0.001 '**' 0.01 '*' 0.05 '.' 0.1 ' ' 1

Residual standard error: 10.11 on 125 degrees of freedom
  (1 observation deleted due to missingness)
Multiple R-squared:  0.5043,   Adjusted R-squared:  0.4924
F-statistic:  42.4 on 3 and 125 DF,  p-value: < 2.2e-16

>
> MultRegVSFemale_DMAx <- lm(d_max ~ Alter + Geschlecht, data = Mistletoe_MorphologyFemaleFirst)
> summary(MultRegVSFemale_DMAx)

Call:
lm(formula = d_max ~ Alter + Geschlecht, data = Mistletoe_MorphologyFemaleFirst)

Residuals:
    Min      1Q  Median      3Q     Max
-18.016  -6.926  -1.064   4.962  27.688

Coefficients:
                       Estimate Std. Error t value Pr(>|t|)
(Intercept)             15.9851     3.2417   4.931 2.55e-06 ***
Alter                    2.5863     0.3034   8.523 4.24e-14 ***
GeschlechtB (male)       0.5588     2.2440   0.249   0.8037
GeschlechtC (juvenile)  -5.6264     3.3399  -1.685   0.0946 .
---
Signif. codes:  0 '***' 0.001 '**' 0.01 '*' 0.05 '.' 0.1 ' ' 1

Residual standard error: 10.11 on 125 degrees of freedom
```

```
  (1 observation deleted due to missingness)
Multiple R-squared:  0.5043,   Adjusted R-squared:  0.4924
F-statistic:  42.4 on 3 and 125 DF,  p-value: < 2.2e-16

>
> ### D acro ###
> MultRegVSJuvenile_DAcro <- lm(d_acro ~ Alter + Geschlecht,
data = Mistletoe_MorphologyJuvenileFirst)
> summary(MultRegVSJuvenile_DAcro)

Call:
lm(formula = d_acro ~ Alter + Geschlecht, data = Mistletoe_MorphologyJuvenileFirst)

Residuals:
    Min      1Q  Median      3Q     Max
-18.867  -6.656  -1.245   4.667  23.791

Coefficients:
                     Estimate Std. Error t value Pr(>|t|)
(Intercept)           12.3368     2.6476   4.660 8.04e-06 ***
Alter                  1.1695     0.2771   4.221 4.66e-05 ***
GeschlechtB (female)   4.6158     3.0509   1.513    0.133
GeschlechtC (male)     1.2708     3.2676   0.389    0.698
---
Signif. codes:  0 '***' 0.001 '**' 0.01 '*' 0.05 '.' 0.1 ' ' 1

Residual standard error: 9.211 on 124 degrees of freedom
  (2 observations deleted due to missingness)
Multiple R-squared:  0.2422,   Adjusted R-squared:  0.2239
F-statistic: 13.21 on 3 and 124 DF,  p-value: 1.531e-07

>
> MultRegVSFemale_DAcro <- lm(d_acro ~ Alter + Geschlecht,
data = Mistletoe_MorphologyFemaleFirst)
> summary(MultRegVSFemale_DAcro)

Call:
lm(formula = d_acro ~ Alter + Geschlecht, data = Mistletoe_MorphologyFemaleFirst)

Residuals:
    Min      1Q  Median      3Q     Max
-18.867  -6.656  -1.245   4.667  23.791

Coefficients:
                       Estimate Std. Error t value Pr(>|t|)
(Intercept)             16.9526     2.9677   5.712 7.80e-08 ***
Alter                    1.1695     0.2771   4.221 4.66e-05 ***
GeschlechtB (male)      -3.3450     2.0487  -1.633    0.105
GeschlechtC (juvenile)  -4.6158     3.0509  -1.513    0.133
---
Signif. codes:  0 '***' 0.001 '**' 0.01 '*' 0.05 '.' 0.1 ' ' 1

Residual standard error: 9.211 on 124 degrees of freedom
  (2 observations deleted due to missingness)
Multiple R-squared:  0.2422,   Adjusted R-squared:  0.2239
F-statistic: 13.21 on 3 and 124 DF,  p-value: 1.531e-07

>
> ### D basi ###
> MultRegVSJuvenile_DBasi <- lm(d_basi ~ Alter + Geschlecht,
data = Mistletoe_MorphologyJuvenileFirst)
> summary(MultRegVSJuvenile_DBasi)
```

```
Call:
lm(formula = d_basi ~ Alter + Geschlecht, data = Mistletoe_MorphologyJuvenileFirst)

Residuals:
    Min      1Q  Median      3Q     Max 
-18.500  -7.338  -1.454   5.218  25.792 

Coefficients:
                     Estimate Std. Error t value Pr(>|t|)    
(Intercept)            12.050      2.840   4.243 4.31e-05 ***
Alter                   1.374      0.298   4.611 9.95e-06 ***
GeschlechtB (female)    3.204      3.279   0.977    0.331    
GeschlechtC (male)      1.786      3.530   0.506    0.614    
---
Signif. codes:  0 '***' 0.001 '**' 0.01 '*' 0.05 '.' 0.1 ' ' 1

Residual standard error: 9.872 on 122 degrees of freedom
  (4 observations deleted due to missingness)
Multiple R-squared:  0.2388,	Adjusted R-squared:  0.2201 
F-statistic: 12.76 on 3 and 122 DF,  p-value: 2.617e-07

> 
> MultRegVSFemale_DBasi <- lm(d_basi ~ Alter + Geschlecht, 
data = Mistletoe_MorphologyFemaleFirst)
> summary(MultRegVSFemale_DBasi)

Call:
lm(formula = d_basi ~ Alter + Geschlecht, data = Mistletoe_MorphologyFemaleFirst)

Residuals:
    Min      1Q  Median      3Q     Max 
-18.500  -7.338  -1.454   5.218  25.792 

Coefficients:
                       Estimate Std. Error t value Pr(>|t|)    
(Intercept)              15.254      3.200   4.767 5.22e-06 ***
Alter                     1.374      0.298   4.611 9.95e-06 ***
GeschlechtB (male)       -1.418      2.230  -0.636    0.526    
GeschlechtC (juvenile)   -3.204      3.279  -0.977    0.331    
---
Signif. codes:  0 '***' 0.001 '**' 0.01 '*' 0.05 '.' 0.1 ' ' 1

Residual standard error: 9.872 on 122 degrees of freedom
  (4 observations deleted due to missingness)
Multiple R-squared:  0.2388,	Adjusted R-squared:  0.2201 
F-statistic: 12.76 on 3 and 122 DF,  p-value: 2.617e-07

> 
> ### D mist ###
> MultRegVSJuvenile_DMist <- lm(d_mist ~ Alter + Geschlecht, 
data = Mistletoe_MorphologyJuvenileFirst)
> summary(MultRegVSJuvenile_DMist)

Call:
lm(formula = d_mist ~ Alter + Geschlecht, data = Mistletoe_MorphologyJuvenileFirst)

Residuals:
    Min      1Q  Median      3Q     Max 
-16.487  -4.219  -1.191   2.097  32.296 

Coefficients:
```

```
                     Estimate Std. Error t value Pr(>|t|)    
(Intercept)           -6.8974     2.4424  -2.824  0.00572 ** 
Alter                  2.9216     0.2988   9.777 3.05e-16 ***
GeschlechtB (female)   1.7367     2.8735   0.604  0.54695    
GeschlechtC (male)    -1.0803     3.0155  -0.358  0.72092    
---
Signif. codes:  0 '***' 0.001 '**' 0.01 '*' 0.05 '.' 0.1 ' ' 1

Residual standard error: 7.878 on 100 degrees of freedom
  (26 observations deleted due to missingness)
Multiple R-squared:  0.6179,   Adjusted R-squared:  0.6065 
F-statistic: 53.91 on 3 and 100 DF,  p-value: < 2.2e-16

> 
> MultRegVSFemale_DMist <- lm(d_mist ~ Alter + Geschlecht, 
data = Mistletoe_MorphologyFemaleFirst)
> summary(MultRegVSFemale_DMist)

Call:
lm(formula = d_mist ~ Alter + Geschlecht, data = Mistletoe_MorphologyFemaleFirst)

Residuals:
    Min      1Q  Median      3Q     Max 
-16.487  -4.219  -1.191   2.097  32.296 

Coefficients:
                       Estimate Std. Error t value Pr(>|t|)    
(Intercept)             -5.1607     3.0954  -1.667   0.0986 .  
Alter                    2.9216     0.2988   9.777 3.05e-16 ***
GeschlechtB (male)      -2.8170     1.9637  -1.435   0.1545    
GeschlechtC (juvenile)  -1.7367     2.8735  -0.604   0.5469    
---
Signif. codes:  0 '***' 0.001 '**' 0.01 '*' 0.05 '.' 0.1 ' ' 1

Residual standard error: 7.878 on 100 degrees of freedom
  (26 observations deleted due to missingness)
Multiple R-squared:  0.6179,   Adjusted R-squared:  0.6065 
F-statistic: 53.91 on 3 and 100 DF,  p-value: < 2.2e-16

> 
> ### L hyp ###
> MultRegVSJuvenile_Lhyp <- lm(l_hyp ~ Alter + Geschlecht, 
data = Mistletoe_MorphologyJuvenileFirst)
> summary(MultRegVSJuvenile_Lhyp)

Call:
lm(formula = l_hyp ~ Alter + Geschlecht, data = Mistletoe_MorphologyJuvenileFirst)

Residuals:
     Min       1Q   Median       3Q      Max 
-12.5385  -3.6996  -0.9186   1.4673  19.2334 

Coefficients:
                     Estimate Std. Error t value Pr(>|t|)    
(Intercept)           -1.1779     1.7888  -0.658    0.512    
Alter                  1.3240     0.1842   7.187  6.5e-11 ***
GeschlechtB (female)   2.7047     2.0564   1.315    0.191    
GeschlechtC (male)     2.8971     2.2118   1.310    0.193    
---
Signif. codes:  0 '***' 0.001 '**' 0.01 '*' 0.05 '.' 0.1 ' ' 1

Residual standard error: 6.053 on 118 degrees of freedom
```

```
  (8 observations deleted due to missingness)
Multiple R-squared:  0.426,	Adjusted R-squared:  0.4114 
F-statistic:  29.2 on 3 and 118 DF,  p-value: 3.424e-14

> 
> MultRegVSFemale_Lhyp <- lm(l_hyp ~ Alter + Geschlecht, 
data = Mistletoe_MorphologyFemaleFirst)
> summary(MultRegVSFemale_Lhyp)

Call:
lm(formula = l_hyp ~ Alter + Geschlecht, data = Mistletoe_MorphologyFemaleFirst)

Residuals:
     Min       1Q   Median       3Q      Max 
-12.5385  -3.6996  -0.9186   1.4673  19.2334 

Coefficients:
                       Estimate Std. Error t value Pr(>|t|)    
(Intercept)              1.5268     1.9598   0.779    0.437    
Alter                    1.3240     0.1842   7.187  6.5e-11 ***
GeschlechtB (male)       0.1924     1.3706   0.140    0.889    
GeschlechtC (juvenile)  -2.7047     2.0564  -1.315    0.191    
---
Signif. codes:  0 '***' 0.001 '**' 0.01 '*' 0.05 '.' 0.1 ' ' 1

Residual standard error: 6.053 on 118 degrees of freedom
  (8 observations deleted due to missingness)
Multiple R-squared:  0.426,	Adjusted R-squared:  0.4114 
F-statistic:  29.2 on 3 and 118 DF,  p-value: 3.424e-14

> 
> ### D max * L hyp ###
> MultRegVSJuvenile_DmaxLHyp <- lm(d_max.l_hyp.1 ~ Alter + Geschlecht, 
data = Mistletoe_MorphologyJuvenileFirst)
> summary(MultRegVSJuvenile_DmaxLHyp)

Call:
lm(formula = d_max.l_hyp.1 ~ Alter + Geschlecht, data = Mistletoe_MorphologyJuvenileFirst)

Residuals:
     Min       1Q   Median       3Q      Max 
-2981145 -1532589 -1088170   239630 23924090 

Coefficients:
                     Estimate Std. Error t value Pr(>|t|)
(Intercept)             33362     960943   0.035    0.972
Alter                   47078      98962   0.476    0.635
GeschlechtB (female)  1042011    1104653   0.943    0.347
GeschlechtC (male)    1961560    1188167   1.651    0.101

Residual standard error: 3252000 on 118 degrees of freedom
  (8 observations deleted due to missingness)
Multiple R-squared:  0.03611,	Adjusted R-squared:  0.0116 
F-statistic: 1.473 on 3 and 118 DF,  p-value: 0.2253

> 
> MultRegVSFemale_DmaxLHyp <- lm(d_max.l_hyp.1 ~ Alter + Geschlecht, 
data = Mistletoe_MorphologyFemaleFirst)
> summary(MultRegVSFemale_DmaxLHyp)

Call:
lm(formula = d_max.l_hyp.1 ~ Alter + Geschlecht, data = Mistletoe_MorphologyFemaleFirst)
```

```
Residuals:
     Min       1Q   Median       3Q      Max 
-2981145 -1532589 -1088170   239630 23924090 

Coefficients:
                       Estimate Std. Error t value Pr(>|t|)
(Intercept)             1075372    1052761   1.021    0.309
Alter                     47078      98962   0.476    0.635
GeschlechtB (male)       919550     736247   1.249    0.214
GeschlechtC (juvenile) -1042011    1104653  -0.943    0.347

Residual standard error: 3252000 on 118 degrees of freedom
  (8 observations deleted due to missingness)
Multiple R-squared:  0.03611,	Adjusted R-squared:  0.0116 
F-statistic: 1.473 on 3 and 118 DF,  p-value: 0.2253

> 
> ### D max / L hyp ###
> MultRegVSJuvenile_Dmax_LHyp <- lm(d_max.l_hyp ~ Alter + Geschlecht, 
data = Mistletoe_MorphologyJuvenileFirst)
> summary(MultRegVSJuvenile_Dmax_LHyp)

Call:
lm(formula = d_max.l_hyp ~ Alter + Geschlecht, data = Mistletoe_MorphologyJuvenileFirst)

Residuals:
    Min      1Q  Median      3Q     Max 
-2.1544 -0.7542 -0.0776  0.4891  3.7549 

Coefficients:
                     Estimate Std. Error t value Pr(>|t|)    
(Intercept)           5.73164    0.33220  17.254  < 2e-16 ***
Alter                -0.09453    0.03421  -2.763 0.006641 ** 
GeschlechtB (female) -1.58569    0.38188  -4.152 6.25e-05 ***
GeschlechtC (male)   -1.64996    0.41075  -4.017 0.000104 ***
---
Signif. codes:  0 ‘***’ 0.001 ‘**’ 0.01 ‘*’ 0.05 ‘.’ 0.1 ‘ ’ 1

Residual standard error: 1.124 on 118 degrees of freedom
  (8 observations deleted due to missingness)
Multiple R-squared:  0.3075,	Adjusted R-squared:  0.2899 
F-statistic: 17.47 on 3 and 118 DF,  p-value: 1.89e-09

> 
> MultRegVSFemale_Dmax_LHyp <- lm(d_max.l_hyp ~ Alter + Geschlecht, 
data = Mistletoe_MorphologyFemaleFirst)
> summary(MultRegVSFemale_Dmax_LHyp)

Call:
lm(formula = d_max.l_hyp ~ Alter + Geschlecht, data = Mistletoe_MorphologyFemaleFirst)

Residuals:
    Min      1Q  Median      3Q     Max 
-2.1544 -0.7542 -0.0776  0.4891  3.7549 

Coefficients:
                       Estimate Std. Error t value Pr(>|t|)    
(Intercept)             4.14595    0.36394  11.392  < 2e-16 ***
Alter                  -0.09453    0.03421  -2.763  0.00664 ** 
GeschlechtB (male)     -0.06427    0.25452  -0.253  0.80108    
GeschlechtC (juvenile)  1.58569    0.38188   4.152 6.25e-05 ***
```

```
---
Signif. codes:  0 ‘***’ 0.001 ‘**’ 0.01 ‘*’ 0.05 ‘.’ 0.1 ‘ ’ 1

Residual standard error: 1.124 on 118 degrees of freedom
  (8 observations deleted due to missingness)
Multiple R-squared:  0.3075,	Adjusted R-squared:  0.2899 
F-statistic: 17.47 on 3 and 118 DF,  p-value: 1.89e-09

> 
> ### D max / D acro ###
> MultRegVSJuvenile_Dmax_DAcro <- lm(d_max.d_acro ~ Alter + Geschlecht,
data = Mistletoe_MorphologyJuvenileFirst)
> summary(MultRegVSJuvenile_Dmax_DAcro)

Call:
lm(formula = d_max.d_acro ~ Alter + Geschlecht, data = Mistletoe_MorphologyJuvenileFirst)

Residuals:
     Min       1Q   Median       3Q      Max 
-0.65365 -0.25722 -0.09331  0.16149  1.98635 

Coefficients:
                     Estimate Std. Error t value Pr(>|t|)    
(Intercept)           1.17646    0.11945   9.849   <2e-16 ***
Alter                 0.02338    0.01250   1.871   0.0638 .  
GeschlechtB (female)  0.13998    0.13764   1.017   0.3111    
GeschlechtC (male)    0.34149    0.14742   2.316   0.0222 *  
---
Signif. codes:  0 ‘***’ 0.001 ‘**’ 0.01 ‘*’ 0.05 ‘.’ 0.1 ‘ ’ 1

Residual standard error: 0.4155 on 124 degrees of freedom
  (2 observations deleted due to missingness)
Multiple R-squared:  0.1093,	Adjusted R-squared:  0.08774 
F-statistic: 5.071 on 3 and 124 DF,  p-value: 0.0024

> 
> MultRegVSFemale_Dmax_DAcro <- lm(d_max.d_acro ~ Alter + Geschlecht,
data = Mistletoe_MorphologyFemaleFirst)
> summary(MultRegVSFemale_Dmax_DAcro)

Call:
lm(formula = d_max.d_acro ~ Alter + Geschlecht, data = Mistletoe_MorphologyFemaleFirst)

Residuals:
     Min       1Q   Median       3Q      Max 
-0.65365 -0.25722 -0.09331  0.16149  1.98635 

Coefficients:
                       Estimate Std. Error t value Pr(>|t|)    
(Intercept)             1.31644    0.13389   9.833   <2e-16 ***
Alter                   0.02338    0.01250   1.871   0.0638 .  
GeschlechtB (male)      0.20150    0.09243   2.180   0.0311 *  
GeschlechtC (juvenile) -0.13998    0.13764  -1.017   0.3111    
---
Signif. codes:  0 ‘***’ 0.001 ‘**’ 0.01 ‘*’ 0.05 ‘.’ 0.1 ‘ ’ 1

Residual standard error: 0.4155 on 124 degrees of freedom
  (2 observations deleted due to missingness)
Multiple R-squared:  0.1093,	Adjusted R-squared:  0.08774 
F-statistic: 5.071 on 3 and 124 DF,  p-value: 0.0024

> 
```

```
> ### D max / D basi ###
> MultRegVSJuvenile_Dmax_DBasi <- lm(d_max.d_basi ~ Alter + Geschlecht,
data = Mistletoe_MorphologyJuvenileFirst)
> summary(MultRegVSJuvenile_Dmax_DBasi)

Call:
lm(formula = d_max.d_basi ~ Alter + Geschlecht, data = Mistletoe_MorphologyJuvenileFirst)

Residuals:
     Min       1Q   Median       3Q      Max
-0.61930 -0.27225 -0.07469  0.19019  1.73070

Coefficients:
                      Estimate Std. Error t value Pr(>|t|)
(Intercept)            1.14643    0.11436  10.025   <2e-16 ***
Alter                  0.01641    0.01200   1.367   0.1741
GeschlechtB (female)   0.22240    0.13206   1.684   0.0947 .
GeschlechtC (male)     0.29545    0.14215   2.079   0.0398 *
---
Signif. codes:  0 '***' 0.001 '**' 0.01 '*' 0.05 '.' 0.1 ' ' 1

Residual standard error: 0.3976 on 122 degrees of freedom
  (4 observations deleted due to missingness)
Multiple R-squared:  0.08658, Adjusted R-squared:  0.06412
F-statistic: 3.855 on 3 and 122 DF,  p-value: 0.01123

>
> MultRegVSFemale_Dmax_DBasi <- lm(d_max.d_basi ~ Alter + Geschlecht,
 data = Mistletoe_MorphologyFemaleFirst)
> summary(MultRegVSFemale_Dmax_DBasi)

Call:
lm(formula = d_max.d_basi ~ Alter + Geschlecht, data = Mistletoe_MorphologyFemaleFirst)

Residuals:
     Min       1Q   Median       3Q      Max
-0.61930 -0.27225 -0.07469  0.19019  1.73070

Coefficients:
                        Estimate Std. Error t value Pr(>|t|)
(Intercept)              1.36883    0.12886  10.623   <2e-16 ***
Alter                    0.01641    0.01200   1.367   0.1741
GeschlechtB (male)       0.07305    0.08979   0.814   0.4175
GeschlechtC (juvenile) -0.22240    0.13206  -1.684   0.0947 .
---
Signif. codes:  0 '***' 0.001 '**' 0.01 '*' 0.05 '.' 0.1 ' ' 1

Residual standard error: 0.3976 on 122 degrees of freedom
  (4 observations deleted due to missingness)
Multiple R-squared:  0.08658, Adjusted R-squared:  0.06412
F-statistic: 3.855 on 3 and 122 DF,  p-value: 0.01123

>
> ### D max / ((D basi + D acro) / 2) ###
> MultRegVSJuvenile_Dmax_DBasi_DAcro <- lm(d_max.d_basi.d_acro ~ Alter + Geschlecht,
data = Mistletoe_MorphologyJuvenileFirst)
> summary(MultRegVSJuvenile_Dmax_DBasi_DAcro)

Call:
lm(formula = d_max.d_basi.d_acro ~ Alter + Geschlecht, data = Mistletoe_MorphologyJuvenileFirst)

Residuals:
```

```
     Min       1Q   Median       3Q      Max 
-0.61651 -0.23747 -0.08683  0.16468  1.87349 

Coefficients:
                     Estimate Std. Error t value Pr(>|t|)    
(Intercept)           1.16001    0.10890  10.652   <2e-16 ***
Alter                 0.01968    0.01145   1.719   0.0882 .  
GeschlechtB (female)  0.17003    0.12602   1.349   0.1798    
GeschlechtC (male)    0.30755    0.13536   2.272   0.0248 *  
---
Signif. codes:  0 '***' 0.001 '**' 0.01 '*' 0.05 '.' 0.1 ' ' 1

Residual standard error: 0.3784 on 121 degrees of freedom
  (5 observations deleted due to missingness)
Multiple R-squared:  0.1041,	Adjusted R-squared:  0.0819 
F-statistic: 4.687 on 3 and 121 DF,  p-value: 0.003925

> 
> MultRegVSFemale_Dmax_DBasi_DAcro <- lm(d_max.d_basi.d_acro ~ Alter + Geschlecht, 
data = Mistletoe_MorphologyFemaleFirst)
> summary(MultRegVSFemale_Dmax_DBasi_DAcro)

Call:
lm(formula = d_max.d_basi.d_acro ~ Alter + Geschlecht, data = Mistletoe_MorphologyFemaleFirst)

Residuals:
     Min       1Q   Median       3Q      Max 
-0.61651 -0.23747 -0.08683  0.16468  1.87349 

Coefficients:
                       Estimate Std. Error t value Pr(>|t|)    
(Intercept)             1.33004    0.12324  10.792   <2e-16 ***
Alter                   0.01968    0.01145   1.719   0.0882 .  
GeschlechtB (male)      0.13752    0.08562   1.606   0.1108    
GeschlechtC (juvenile) -0.17003    0.12602  -1.349   0.1798    
---
Signif. codes:  0 '***' 0.001 '**' 0.01 '*' 0.05 '.' 0.1 ' ' 1

Residual standard error: 0.3784 on 121 degrees of freedom
  (5 observations deleted due to missingness)
Multiple R-squared:  0.1041,	Adjusted R-squared:  0.0819 
F-statistic: 4.687 on 3 and 121 DF,  p-value: 0.003925




> 
```
